# Products of two atoms in Krull monoids and arithmetical characterizations of class groups^☆^

**DOI:** 10.1016/j.ejc.2013.05.008

**Published:** 2013-11

**Authors:** Paul Baginski, Alfred Geroldinger, David J. Grynkiewicz, Andreas Philipp

**Affiliations:** a Department of Mathematics and Statistics, Smith College, Northampton, MA 01063, USA; b Institut für Mathematik und Wissenschaftliches Rechnen, Karl-Franzens-Universität Graz, Heinrichstraße 36, 8010 Graz, Austria

## Abstract

Let H be a Krull monoid with finite class group G such that every class contains a prime divisor and let D(G) be the Davenport constant of G. Then a product of two atoms of H can be written as a product of at most D(G) atoms. We study this extremal case and consider the set $U_{\left\{2,D\left(G\right)\right\}}\left(H\right)$ defined as the set of all l∈N with the following property: there are two atoms u,v∈H such that uv can be written as a product of l atoms as well as a product of D(G) atoms. If G is cyclic, then $U_{\left\{2,D\left(G\right)\right\}}\left(H\right)=\left\{2,D\left(G\right)\right\}$. If G has rank two, then we show that (apart from some exceptional cases) $U_{\left\{2,D\left(G\right)\right\}}\left(H\right)=\left[2,D\left(G\right)\right]\setminus \left\{3\right\}$. This result is based on the recent characterization of all minimal zero-sum sequences of maximal length over groups of rank two. As a consequence, we are able to show that the arithmetical factorization properties encoded in the sets of lengths of a rank 2 prime power order group uniquely characterizes the group.

*Dedicated to the memory of our friend and colleague Yahya ould Hamidoune*

## 1 Introduction

Let H be a Krull monoid with finite class group G and suppose that every class contains a prime divisor (rings of integers in algebraic number fields are such Krull monoids, and other examples will be given in Section  2). Then every non-unit a∈H can be written as a finite product of atoms (irreducible elements), say $a=u_{1}\cdot \ldots \cdot u_{k}$, and the number k of atoms is called the length of the factorization. The set L(a)⊂N of all possible k is called the set of lengths of a, and it is easy to argue that L(a) is finite. It is well-known that H is factorial if and only if |G|=1, and that H is half-factorial (this means |L(a)|=1 for all non-units a∈H) if and only if |G|≤2. Suppose that |G|≥3. Then there exists an a∈H with |L(a)|>1, and therefore, for every N∈N, there is an $a_{N}\in H$ with $\left|L\left(a_{N}\right)\right|>N$ (indeed, $a^{N}$ has this property).

Long sets of lengths have a well-defined structure: they are AAMPs (almost arithmetical multiprogressions) with a universal bound for all parameters [17, Chapter 4], and this description is the best possible [31]. For every k∈N, let $U_{k}\left(H\right)$ denote the set of all l∈N such that a product of k atoms can be written as a product of l atoms (by definition, $U_{k}\left(H\right)$ is the union of all sets of lengths L(a) with k∈L(a)). It is not difficult to show that these unions $U_{k}\left(H\right)$—first studied in [5]—are intervals [14, Theorem 3.1.3]. Their maxima are $\rho_{k}\left(H\right)$, i.e.,  $\rho_{k}\left(H\right)=\max U_{k}\left(H\right)$, which, like the elasticity $\rho \left(H\right)=\sup\left\{\rho_{l}\left(H\right)/l|l\in N\right\}$, are widely studied invariants. An easy observation shows that $\rho_{k}\left(H\right)\leq kD\left(G\right)/2$, where D(G) is the Davenport constant of G, and that equality holds for even k[17, Section 6.3]. The question for the precise value of $\rho_{k}\left(H\right)$ for odd k is settled for cyclic groups [11] but open in general [16].

Little is known about short sets of lengths. If u,v∈H are two atoms, then maxL(uv)≤D(G), and we will consider the extremal case where this maximum is attained. More precisely, we study the set $U_{\left\{2,D\left(G\right)\right\}}\left(H\right)$ which is defined as the set of all l∈N with the following property:

There are two atoms u,v∈H such that uv can be written as a product of l atoms as well as a product of D(G) atoms.

Thus $U_{\left\{2,D\left(G\right)\right\}}\left(H\right)$ is the union of all sets of lengths L(a) with {2,D(G)}⊂L(a), and we have $\left\{2,D\left(G\right)\right\}\subset U_{\left\{2,D\left(G\right)\right\}}\left(H\right)\subset \left[2,D\left(G\right)\right]$. Our starting point is the following result [17, Theorem 6.6.3].

Theorem A *Let* H *be a Krull monoid with finite class group* G,|G|≥3 *, and suppose that every class contains a prime divisor. Then*

$$U_{\left\{2,D\left(G\right)\right\}}\left(H\right)=\left\{2,D\left(G\right)\right\}\;\text{if and only if}\;G\text{ is cyclic or an elementary }2\text{-group .}$$

Our first main result (Theorem 3.5) shows that, in groups of rank two, $U_{\left\{2,D\left(G\right)\right\}}\left(H\right)$ equals [2,D(G)]∖{3} (apart from some exceptional cases). We extend this to groups of higher rank (Theorem 4.2), and these two results are the key for a characterization result on class groups (Theorem 5.6; the status on arithmetical characterizations of class groups will be discussed at the beginning of Section  5).

It is well known that all questions on sets of lengths in a Krull monoid translate into zero-sum problems in its class group. Thus, after applying well-studied transfer machinery (Lemma 2.1), all the algebraic problems outlined above turn out to be combinatorial ones. Indeed, the present progress is entirely based on the characterization of all minimal zero-sum sequences of maximal length over groups of rank two (see Theorem 3.1). The characterization result (Theorem 5.6) substantially uses recent work by Schmid [31], [30], [33].

## 2 Preliminaries

Let N denote the set of positive integers, P⊂N the set of prime numbers and put $N_{0}=N\cup \left\{0\right\}$. For real numbers a,b∈R, we set [a,b]={x∈Z∣a≤x≤b}. For subsets A,B⊂Z, we denote by A+B={a+b∣a∈A,b∈B} their *sumset*, and by Δ(A) the *set of (successive) distances* of A (that is, d∈Δ(A) if and only if d=b−a with a,b∈A distinct and [a,b]∩A={a,b}).

Let G be an additively written finite abelian group and $G_{0}\subset G$ a subset. Then $\left[G_{0}\right]\subset G$ denotes the sub-semigroup generated by $G_{0}$, and $\langle G_{0}\rangle \subset G$ denotes the subgroup generated by $G_{0}$. A tuple ${\left(e_{i}\right)}_{i\in I}$ of elements of G is said to be *independent* if all elements are non-zero and

$$\underset{i\in I}{\sum }m_{i}e_{i}=0\;\text{implies }m_{i}e_{i}=0\text{ for all }i\in I,\text{ where }m_{i}\in Z\text{.}$$

The tuple ${\left(e_{i}\right)}_{i\in I}$ is called a *basis* if ${\left(e_{i}\right)}_{i\in I}$ is independent and $\langle \left\{e_{i}|i\in I\right\}\rangle =G$. For p∈P, let $r_{p}\left(G\right)$ denote the p-rank of $G,r\left(G\right)=\max\left\{r_{p}\left(G\right)|p\in P\right\}$ denote the rank of G, and let $r^{*}\left(G\right)=\sum_{p\in P}r_{p}\left(G\right)$ be the *total rank* of G. For n∈N, let $C_{n}$ denote a cyclic group with n elements. If |G|>1, then we have

$$G≅C_{n_{1}}⊕\cdots ⊕C_{n_{r}}\text{,}\;\text{and we set }d^{*}\left(G\right)=\overset{r}{\underset{i=1}{\sum }}\left(n_{i}-1\right)\;\text{and}\;D^{*}\left(G\right)=d^{*}\left(G\right)+1\text{,}$$

where $r=r\left(G\right)\in N,n_{1},\ldots ,n_{r}\in N$ are integers with $1<n_{1}|\ldots |n_{r}$ and $n_{r}=\exp\left(G\right)$ is the exponent of G. If g∈G with ord(g)=exp(G), then there exist $e_{1},\ldots ,e_{r-1}\in G$ with $\mathrm{ord}\left(e_{i}\right)=n_{i}$ for all i∈[1,r−1] such that $\left(e_{1},\ldots ,e_{r-1},g\right)$ is a basis of G. If |G|=1, then $r\left(G\right)=0,\exp\left(G\right)=1,d^{*}\left(G\right)=0$, and $D^{*}\left(G\right)=1$.

*Monoids and factorizations.* By a *monoid*, we always mean a commutative semigroup with identity which satisfies the cancellation law (that is, if a,b,c are elements of the monoid with ab=ac, then b=c follows). The multiplicative semigroup of non-zero elements of an integral domain is a monoid. Let H be a monoid. We denote by $H^{\times }$ the set of invertible elements of H, and we say that H is  *reduced*  if $H^{\times }=\left\{1\right\}$. Let q(H) be a quotient group and A(H) the set of atoms of H. Let $a\in H\setminus H^{\times }$. If $a=u_{1}\cdot \ldots \cdot u_{k}$, with $u_{1},\ldots ,u_{k}\in A\left(H\right)$, then k is called the length of the factorization, and the set $L_{H}\left(a\right)=L\left(a\right)\subset N$ of all possible k is called the *set of lengths* of a (with respect to the monoid H). For convenience, we set L(a)={0} for $a\in H^{\times }$. We denote by

L(H)={L(a)∣a∈H}  the  system of sets of lengths  of  H,  and by

$$\Delta \left(H\right)=\underset{L\in L\left(H\right)}{⋃}\Delta \left(L\right)\subset N\;\text{the set of distances of }H\text{.}$$

If $H\neq H^{\times }$ and M⊂N is a subset, we set

$$U_{M}\left(H\right)=\underset{M\subset L,L\in L\left(H\right)}{⋃}L\text{,}$$

which is the union of all sets of lengths containing M. In the case |M|=1, these unions are well studied (see for example  [5], [6], [2]).

A monoid F is called  *free* (*abelian, with basis* P⊂F) if every a∈F has a unique representation of the form

$$a=\underset{p\in P}{\prod }p^{v_{p}\left(a\right),}\;\text{where }v_{p}\left(a\right)\in N_{0}\;\text{ with }v_{p}\left(a\right)=0\text{ for almost all }p\in P\text{.}$$

We set F=F(P) and call

$${\left|a\right|}_{F}=\left|a\right|=\underset{p\in P}{\sum }v_{p}\left(a\right)\;\text{the length of }a\text{.}$$

*Krull monoids.* The theory of Krull monoids is presented in the monographs  [23], [17]. We briefly summarize what is needed in what follows. The monoid H is called a *Krull monoid* if it satisfies one of the following equivalent conditions [17, Theorem 2.4.8].

• H is v-noetherian and completely integrally closed.
• H has a divisor theory. This means that there is a monoid homomorphism φ:H→D=F(P) into a free monoid with the following properties:
  – For every a,b∈H,φ(a)∣φ(b) implies that a∣b.
  – For every p∈P, there exists a finite subset 0̸≠X⊂H such that gcd(φ(X))=p.

Let H be a Krull monoid. Then a divisor theory φ:H→D is essentially unique, and the group C(H)=q(D)/q(φ(H))—called the *class group* of H—does indeed depend only on H. It will be written additively, and the set

$$G_{P}=\left\{\left[p\right]=pq\left(\varphi \left(H\right)\right)|p\in P\right\}\subset C\left(H\right)$$

is called the *set of classes containing prime divisors*. We have $\left[G_{P}\right]=C\left(H\right)$.

An integral domain R is a Krull domain if and only if its multiplicative monoid R∖{0} is a Krull monoid, and a noetherian domain is Krull if and only if it is integrally closed. Rings of integers, holomorphy rings in algebraic function fields, and regular congruence monoids in these domains are Krull monoids with finite class group such that every class contains a prime divisor [17, Section 2.11]. Monoid domains and power series domains that are Krull and have prime divisors in all classes are discussed in  [24], [25], [3].

Main portions of the arithmetic of a Krull monoid—in particular, all questions dealing with sets of lengths—can be studied in the associated block monoid over its class group. We first provide these concepts and summarize the connection in Lemma 2.1.

*Zero-sum sequences.* Let $G_{0}\subset G$ be a subset. For our purposes, it is convenient to consider sequences over $G_{0}$ as elements in the free monoid $F\left(G_{0}\right)$. Thus sequences will be written multiplicatively. For such a sequence

$$S=g_{1}\cdot \ldots \cdot g_{l}=\underset{g\in G_{0}}{\prod }g^{v_{g}\left(S\right)}\in F\left(G_{0}\right)\text{,}$$

we set $\varphi \left(S\right)=\varphi \left(g_{1}\right)\cdot \ldots \cdot \varphi \left(g_{l}\right)$ for any homomorphism $\varphi :G\to G^{\prime }$, and in particular, we have $-S=\left(-g_{1}\right)\cdot \ldots \cdot \left(-g_{l}\right)$. We call $v_{g}\left(S\right)$ the *multiplicity* of g in S,

$$\left|S\right|=l=\underset{g\in G}{\sum }v_{g}\left(S\right)\in N_{0}\text{ the length of }S\text{,}\;\mathrm{supp}\left(S\right)=\left\{g\in G|v_{g}\left(S\right)>0\right\}\subset G\;\text{the support of }S\text{,}$$

$$\sigma \left(S\right)=\overset{l}{\underset{i=1}{\sum }}g_{i}\text{ the sum of }S\;\text{and}\;\Sigma \left(S\right)=\left\{\underset{i\in I}{\sum }g_{i}|0̸\neq I\subset \left[1,l\right]\right\}\text{ the set of subsequence sums of }S\text{.}$$

The sequence S is said to be

• *zero-sum free* if 0∉Σ(S),
• a *zero-sum sequence* if σ(S)=0,
• a *minimal zero-sum sequence* if it is a nontrivial zero-sum sequence and every proper subsequence is zero-sum free.

The monoid $B\left(G_{0}\right)=\left\{S\in F\left(G_{0}\right)|\sigma \left(S\right)=0\right\}$ is called the *monoid of zero-sum sequences* over $G_{0}$, and we have $B\left(G_{0}\right)=B\left(G\right)\cap F\left(G_{0}\right)$. It is a Krull monoid, and its atoms are precisely the minimal zero-sum sequences.

For every arithmetical invariant ∗(H) defined for a monoid H, it is usual to write $*\left(G_{0}\right)$ instead of $*\left(B\left(G_{0}\right)\right)$ (although this is an abuse of language, there will be no danger of confusion). In particular, we set   $A\left(G_{0}\right)=A\left(B\left(G_{0}\right)\right),L\left(G_{0}\right)=L\left(B\left(G_{0}\right)\right)$, and $U_{M}\left(G_{0}\right)=U_{M}\left(B\left(G_{0}\right)\right)$ for a subset M⊂N. The *Davenport constant*

$$D\left(G_{0}\right)=\max\left\{\left|U\right|\;|\;U\in A\left(G_{0}\right)\right\}\in N_{0}$$

is a classical constant in Combinatorial Number Theory (see the surveys  [10], [14], or  [19], [34], [7] for recent progress). We denote by d(G) the maximal length of a zero-sum free sequence, and get

(2.1) $$1+d^{*}\left(G\right)\leq 1+d\left(G\right)=D\left(G\right)\text{.}$$

We will use without further mention that equality holds if G is a p-group or r(G)≤2 (see  [17, Chapter 5] and  [14, Section 4.2]).

Lemma 2.1 *Let* H *be a Krull monoid,* φ:H→F=F(P) *a divisor theory with some nonempty set* P,G *its class group, and* $G_{P}\subset G$ *the set of classes containing prime divisors. Let* $\overset{˜}{\beta }:F\to F\left(G_{P}\right)$ *denote the unique homomorphism defined by* $\overset{˜}{\beta }\left(p\right)=\left[p\right]$ *for all* p∈P *. Then* $B\left(G_{P}\right)$ *is called the block monoid associated to* H *, and the homomorphism* $\beta =\overset{˜}{\beta }\circ \varphi :H\to B\left(G_{P}\right)$ *has the following property:*

$$L_{H}\left(a\right)=L_{B\left(G_{P}\right)}\left(\beta \left(a\right)\right)\;\text{for every }a\in H\text{.}$$

*This implies that* $L\left(H\right)=L\left(G_{P}\right)$ *and* $U_{M}\left(H\right)=U_{M}\left(G_{P}\right)$ *for every* M⊂N *.*

Proof See  [17, Theorem 3.4.10]. □

The following simple technical lemma will be used without further mention.

Lemma 2.2 *Let* G *be a finite abelian group and* U,V∈A(G∖{0}) *.*

1. maxL(UV)≤min{|U|,|V|}≤D(G) *, and* maxL(UV)=D(G) *if and only if* V=−U *and* |U|=D(G) *.*
2. *If* V∣(−U)U *, then* 2+|U|−|V|∈L((−U)U) *. In particular, if* g∈G *with* $g^{\mathrm{ord}\left(g\right)-1}|U$ *, then* ord(g)∈L((−U)U) *.*

Proof See  [17, Lemmas 6.4.4 and 6.4.5]. □

## 3 Products of two atoms in Krull monoids with class group of rank two

The following characterization of minimal zero-sum sequences of maximal length over groups of rank two—formulated in Theorem 3.1—will be crucial for the present paper. The characterization was achieved by contributions of many authors including Bhowmik, Gao, Halupczok, Reiher, Schlage-Puchta, Schmid, and the second and third authors of the present article [9], [1], [12], [32], [27]. We have reworded the description of type II so that it is described in terms of a basis, rather than a generating set. This alternative description is routinely derived from the original formulation using the fact, previously mentioned, that in a rank 2 group, any element of maximal order exp(G) can always be paired with another existent element to form a basis. We have also made the description of type II slightly stronger, in order to minimize the overlap between sequences described by type I and those described by type II.

Theorem 3.1 *Let* $G=C_{m}⊕C_{mn}$ *with* m,n∈N *and* m≥2 *. A sequence* S *over* G *of length* D(G)=m+mn−1 *is a minimal zero-sum sequence if and only if it has one of the following two forms:*

• $$S=e_{1}^{\mathrm{ord}\left(e_{1}\right)-1}\overset{\mathrm{ord}\left(e_{2}\right)}{\underset{i=1}{\prod }}\left(x_{i}e_{1}+e_{2}\right)$$
  *where*
  (a) $\left(e_{1},e_{2}\right)$*is a basis of*G*,*
  (b) $x_{1},\ldots ,x_{\mathrm{ord}\left(e_{2}\right)}\in \left[0,\mathrm{ord}\left(e_{1}\right)-1\right]$*and*$x_{1}+\cdots +x_{\mathrm{ord}\left(e_{2}\right)}\equiv 1\mathrm{mod}\mathrm{ord}\left(e_{1}\right)$*.*
  *In this case, we say that*S*is of type*  I*.*
• $$S={\left(e_{1}+ye_{2}\right)}^{sm-1}e_{2}^{\left(n-s\right)m+\epsilon }\overset{m-\epsilon }{\underset{i=1}{\prod }}\left(-x_{i}e_{1}+\left(-x_{i}y+1\right)e_{2}\right)\text{,}$$
  *where*
  (a) $\left(e_{1},e_{2}\right)$*is a basis of*G*with*$\mathrm{ord}\left(e_{1}\right)=m$*and*$\mathrm{ord}\left(e_{2}\right)=mn$*,*
  (b) y∈[0,mn−1],ϵ∈[1,m−1]*, and*s∈[1,n−1]*,*
  (c) $x_{1},\ldots ,x_{m-\epsilon }\in \left[1,m-1\right]$*with*$x_{1}+\cdots +x_{m-\epsilon }=m-1$*,*
  (d) $mye_{2}\neq 0$*, and*
  (e) *either*s=1*or*$mye_{2}=me_{2}$*.*
  *In this case, we say that*S*is of type*  II*.*

Proof See the corollary in  [12, p. 104]. Apart from [12], the Corollary is based on  [32], and its assumption is satisfied by  [27]. In the original formulation, it was also allowed that s=n in type II and (d) was not included. We provide a short explanation here as to why, in both these cases, we instead fall under the hypotheses of type I.

If s=n, then $e_{1}^{\prime }≔e_{1}+ye_{2}$ is an element of multiplicity mn−1=exp(G)−1, and thus we must have $\mathrm{ord}\left(e_{1}^{\prime }\right)=mn$ (else S will not be a minimal zero-sum sequence). In this case, as previously mentioned, there is some $e_{2}^{\prime }\in G$, with $\mathrm{ord}\left(e_{2}^{\prime }\right)=m$, such that $\left(e_{1}^{\prime },e_{2}^{\prime }\right)$ gives a basis of G. We can then write $S={e_{1}^{\prime }}^{mn-1}T$ with $T=\prod_{i=1}^{m}\left(y_{i}e_{1}^{\prime }+z_{i}e_{2}^{\prime }\right)$ and $y_{i},\;z_{i}\in \left[0,mn-1\right]$. Let $H=\langle e_{1}^{\prime }\rangle$. Since $\Sigma^{*}\left({e_{1}^{\prime }}^{mn-1}\right)=H$, any proper zero-sum modulo H subsequence of T can be extended to a proper zero-sum subsequence of S, contradicting that S∈A(G). Thus $\phi_{H}\left(T\right)$ must be a minimal zero-sum sequence in $G/H≅C_{m}$. Since $\left|\phi_{H}\left(T\right)\right|=m=\left|G/H\right|=D\left(G/H\right)$, the characterization  [17, Theorem 5.1.10.1] of such sequences implies that all terms of $\phi_{H}\left(T\right)$ are equal to a generating element, which allows us to assume $z_{i}=z_{j}=z$ for all i,j∈[1,m] with $\mathrm{ord}\left(ze_{2}^{\prime }\right)=m$. But now, we see that S also has type I, as desired.

If $mye_{2}=0$, then $\mathrm{ord}\left(e_{1}+ye_{2}\right)=m$, so that $\left(e_{1}+ye_{2},e_{2}\right)$ is a basis of G. Moreover, since (b) implies s∈[1,n−1], we have n≥2, whence (a) gives $\mathrm{ord}\left(e_{2}\right)=mn>m$. Thus $me_{2}\neq 0=mye_{2}$, so that (e) implies s=1. But now it is easily seen that S also has type I, as desired. □

Lemma 3.2 *Let* $G=C_{m}⊕C_{mn}$ *with* m,n∈N *and* m≥2 *.*

1. *We have* $\left\{2,m,mn,D\left(G\right)\right\}\subset U_{\left\{2,D\left(G\right)\right\}}\left(G\right)$ *.*
2. *If* m=2 *, then* {L∈L(G)∣{2,D(G)}⊂L}={{2,2a,2n,2n+1}∣a∈[1,n]} *.*

Proof 1. This follows immediately from the special case in Lemma 2.2.2 and from the (easy direction of) Theorem 3.1.

2. For n=1, the statement is obvious. Suppose that n≥2. If L∈L(G) with {2,D(G)}⊂L, then L=L((−U)U) with U∈A(G) and |U|=D(G). Furthermore, there exists a basis $\left(e_{1},e_{2}\right)$ of G with $\mathrm{ord}\left(e_{1}\right)=2$ and $\mathrm{ord}\left(e_{2}\right)=2n$ such that U has one of the forms given in Case 1 or in Case 2 (this can be seen by a careful analysis of Theorem 3.1 for m=2, or directly from [8, Corollary 3.4]).

*Case* 1. $U=e_{1}e_{2}^{v}{\left(e_{1}+e_{2}\right)}^{2n-v}$ with v∈[3,2n−3] odd.

We set $V_{1}=e_{1}\left(-e_{2}\right)\left(e_{1}+e_{2}\right),V_{2}=\left(e_{1}+e_{2}\right)\left(e_{1}-e_{2}\right)$ and $V_{3}=e_{2}\left(-e_{2}\right)$. If V∈A(G) with $\left(e_{1}+e_{2}\right)|V|\left(-U\right)U$, then $V\in \left\{V_{1},V_{2},U\right\}$. This implies that $\left(-U\right)U=V_{1}\left(-V_{1}\right)V_{2}^{2n-v-1}V_{3}^{v-1}$ is the only factorization of length l∈[3,D(G)−1], and clearly we have l=2n.

*Case* 2. $U=e_{2}^{2n-1}\left(e_{1}+ae_{2}\right)\left(e_{1}+\left(1-a\right)e_{2}\right)$ with a∈[0,2n−1].

Let a∈[0,2n−1] and suppose that

$$\left(-U\right)U=V_{1}\cdot \ldots \cdot V_{l}\;\text{where }V_{1},\ldots ,V_{l}\in A\left(G\right)\;\text{with}\;\left|V_{2}\right|\geq \cdots \geq \left|V_{l}\right|\;\text{and}\;\left(e_{1}+ae_{2}\right)|V_{1}\text{.}$$

If $V_{1}=\left(e_{1}+ae_{2}\right)\left(e_{1}-ae_{2}\right)$, then—up to renumbering if necessary—$V_{2}=\left(e_{1}+\left(1-a\right)e_{2}\right)\left(e_{1}+\left(a-1\right)e_{2}\right),V_{3}=\cdots =V_{l}=\left(-e_{2}\right)e_{2}$, and hence l=2n+1.

If $\left(e_{1}+ae_{2}\right)\left(e_{1}+\left(1-a\right)e_{2}\right)|V_{1}$, then either $V_{1}=U$, $V_{2}=-U$ and l=2, or $V_{1}=\left(e_{1}+ae_{2}\right)\left(e_{1}+\left(1-a\right)e_{2}\right)\left(-e_{2}\right),V_{2}=-V_{1},V_{3}=\cdots =V_{l}=\left(-e_{2}\right)e_{2}$, and hence l=2n.

Suppose that $\left(e_{1}+ae_{2}\right)\left(e_{1}+\left(a-1\right)e_{2}\right)|V_{1}$, and let $b_{1},b_{2}\in \left[0,2n-1\right]$ be such that

$$2a-1+b_{1}\equiv 0\mathrm{mod}2n\;\text{and}\;2a-1-b_{2}\equiv 0\mathrm{mod}2n\text{.}$$

Then either $V_{1}=\left(e_{1}+ae_{2}\right)\left(e_{1}+\left(a-1\right)e_{2}\right)e_{2}^{b_{1}},V_{2}=-V_{1}$, $V_{3}=\cdots =V_{l}=\left(-e_{2}\right)e_{2}$ and hence $l=2n+1-b_{1}\equiv 0\mathrm{mod}2$, or $V_{1}=\left(e_{1}+ae_{2}\right)\left(e_{1}+\left(a-1\right)e_{2}\right){\left(-e_{2}\right)}^{b_{2}},V_{2}=-V_{1},V_{3}=\cdots =V_{l}=\left(-e_{2}\right)e_{2}$ and hence $l=2n+1-b_{2}\equiv 0\mathrm{mod}2$. It is easy to see that all even lengths between 2 and 2n are actually obtained. □

Lemma 3.3 *Let* $G=G_{1}⊕C_{m}⊕C_{mn}$ *, where* $G_{1}\subset G$ *is a possibly trivial subgroup and* m,n∈N *,* m≥2 *, such that* $d^{*}\left(G\right)=d^{*}\left(G_{1}\right)+d^{*}\left(C_{m}⊕C_{mn}\right)$ *. Then there exists some* L∈L(G) *with* $\left\{2\right\}\cup \left[d^{*}\left(G_{1}\right)+mn,D^{*}\left(G\right)\right]\subset L$ *.*

Proof Let $e_{1},e_{2}\in G$ with $\mathrm{ord}\left(e_{1}\right)=m,\mathrm{ord}\left(e_{2}\right)=mn$ and $G=G_{1}⊕\langle e_{1}\rangle ⊕\langle e_{2}\rangle$. Furthermore, let S be a zero-sum free sequence over $G_{1}$ of length $\left|S\right|=d^{*}\left(G_{1}\right)$, and choose k∈[0,m−1] such that $k\equiv 2-\frac{1}{2}m\left(m-1\right)\mathrm{mod}m$. We define

$$U=Se_{1}^{m-1}e_{2}^{mn-m+1}\left(ke_{1}+e_{2}-\sigma \left(S\right)\right)\overset{m-1}{\underset{\nu =2}{\prod }}\left(\nu e_{1}+e_{2}\right)\text{.}$$

Then $\left|U\right|=D^{*}\left(G\right),{\left(ke_{1}+e_{2}-\sigma \left(S\right)\right)}^{-1}U$ is zero-sum free, and since

$$k+\overset{m-1}{\underset{\nu =2}{\sum }}\nu =\left(k-1\right)+\frac{m\left(m-1\right)}{2}\equiv 2-\frac{m\left(m-1\right)}{2}-1+\frac{m\left(m-1\right)}{2}\equiv 1\mathrm{mod}m\text{,}$$

it follows that U∈A(G). We set $V_{1}={\left(-e_{1}\right)}^{m-1}\left(-e_{1}+e_{2}\right)\left(-e_{2}\right)$ and, for every i∈[2,m−1], we set $V_{i}=e_{1}^{m-i}\left(ie_{1}+e_{2}\right)\left(-e_{2}\right)$. Then, for every $i\in \left[1,m-1\right],V_{i}\in A\left(G\right)$ is a divisor of (−U)U of length $\left|V_{i}\right|=m-i+2$. Since $2+\left|U\right|-\left|V_{i}\right|=d^{*}\left(G_{1}\right)+mn+i-1$, the assertion follows from Lemma 2.2. □

The following proposition is one of the more lengthy and difficult portions of the paper.

Proposition 3.4 *Let* $G=C_{m}⊕C_{mn}$ *with* m,n∈N *and* m≥5 *, and let* U∈A(G) *with* |U|=D(G) *. Then* 3∉L((−U)U) *.*

Proof Per Theorem 3.1, there are two main possibilities for the structure of U. We handle these cases separately.

*Case* 1. U has type I in Theorem 3.1.

Then there is a basis of G, say $\left(e_{1},e_{2}\right)$ with $\mathrm{ord}\left(e_{1}\right)=n_{1}$ and $\mathrm{ord}\left(e_{2}\right)=n_{2}$, such that $U=e_{1}^{n_{1}-1}\prod_{i=1}^{n_{2}}\left(x_{i}e_{1}+e_{2}\right)$ with $\sum_{i=1}^{n_{2}}x_{i}\equiv 1\mathrm{mod}n_{1}$. If $n_{2}<m$, then, since $n_{1}n_{2}=\left|G\right|=m^{2}n$, it would follow that $n_{1}>mn$. But this would mean $e_{2}$ was an element with $\mathrm{ord}\left(e_{2}\right)>mn=\exp\left(G\right)$, which is not possible. Therefore we conclude that

$$n_{2}\geq m\geq 5\text{.}$$

Likewise, $n_{1}\geq m\geq 5$. We continue with the following assertion.

**A.** Let $V=e_{1}^{n_{1}-k}\prod_{i\in I}\left(x_{i}e_{1}+e_{2}\right)\prod_{i\in J}\left(-x_{i}e_{1}-e_{2}\right)\in A\left(G\right)$ with $k\in \left[1,n_{1}\right],V|U\left(-U\right),V\neq U$ and $V\neq e_{1}\prod_{i=1}^{n_{2}}\left(-x_{i}e_{i}-e_{2}\right)$. Then $\left|I\right|=\left|J\right|\leq \min\left\{k,n_{2}\right\}$.

Proof of A Since V∣(−U)U, clearly $\left|I\right|,\left|J\right|\leq n_{2}$. If V is trivial, then clearly |I|=|J|=0≤k holds. So we may assume V is nontrivial.

Since V is a zero-sum sequence, its sum must have zero as its $e_{2}$-coordinate. Thus either |I|=|J| or $\left|I\right|,\;\left|J\right|\in \left\{0,\;n_{2}\right\}$. Suppose the latter occurs. If |J|=0, then V is a nontrivial subsequence of the minimal zero-sum sequence U, whence V=U, contrary to hypothesis. Therefore $\left|J\right|=n_{2}$. If $\left|I\right|=n_{2}$, then V will contain $n_{2}\geq 2$ nontrivial, zero-sum subsequences of the form $\left(x_{i}e_{1}+e_{2}\right)\left(-x_{i}e_{1}-e_{2}\right)$, contradicting that V is assumed to be an atom. Therefore |I|=0. But now, since $\sum_{i=1}^{n_{2}}x_{i}\equiv 1\mathrm{mod}n_{1}$ with |I|=0 and $J=\left[1,n_{2}\right]$, we easily deduce that $V=e_{1}\prod_{i=1}^{n_{2}}\left(-x_{i}e_{i}-e_{2}\right)$, contrary to hypothesis. So we instead conclude that |I|=|J| must hold.

Since V is a zero-sum sequence, we have $\sum_{i\in I}x_{i}+\sum_{i\in J}\left(-x_{i}\right)\equiv k\mathrm{mod}n_{1}$. Write $\left|I\right|=\left|J\right|=l,I=\left\{i_{1},\ldots ,i_{l}\right\}$, and $J=\left\{j_{1},\ldots ,j_{l}\right\}$. Now we find $\sum_{q=1}^{l}\left(x_{i_{q}}-x_{j_{q}}\right)\equiv k\mathrm{mod}n_{1}$.

If, for some $q\in \left[1,l\right],x_{i_{q}}=x_{j_{q}}$, then $\left(x_{i_{q}}e_{1}+e_{2}\right)\left(-x_{j_{q}}e_{1}-e_{2}\right)$ is a non-trivial zero-sum subsequence of V. Thus, since V is an atom, this is only possible if $V=\left(x_{i_{q}}e_{1}+e_{2}\right)\left(-x_{j_{q}}e_{1}-e_{2}\right)$, in which case |I|=|J|=1≤k, as desired. Therefore we may assume $x_{i_{q}}\neq x_{j_{q}}$ for all q∈[1,l].

Assume by contradiction that l>k. Consider the partial sums $\sum_{q=1}^{r}\left(x_{i_{q}}-x_{j_{q}}\right)$ for r∈[1,l]. If 2 of these sums were equal modulo $n_{1}$, then the terms contained in the longer sum but not the shorter sum would sum to zero modulo $n_{1}$, corresponding to a proper, nontrivial zero-sum subsequence of V, contradicting that V is an atom. As a result, we conclude that sums $\sum_{q=1}^{r}\left(x_{i_{q}}-x_{j_{q}}\right)$, for r∈[1,l], are distinct modulo $n_{1}$. Consequently, since l≥k+1, it follows that there is some nonempty subset M⊂[1,l] such that $\sum_{q\in M}\left(x_{i_{q}}-x_{j_{q}}\right)\equiv k^{\prime }\mathrm{mod}n_{1}$ with $k^{\prime }\in \left[k+1,n_{1}\right]$. Moreover, since $\sum_{q\in \left[1,l\right]}\left(x_{i_{q}}-x_{j_{q}}\right)\equiv k\mathrm{mod}n_{1}$ as noted above, we see that M⊂[1,l] must be a proper subset. But this leads to a proper, non-trivial zero-sum subsequence

$$e_{1}^{n_{1}-k^{\prime }}\underset{q\in M}{\prod }\left(x_{i_{q}}e_{1}+e_{2}\right)\left(-x_{j_{q}}e_{1}-e_{2}\right)|V\text{,}$$

once more contradicting that V is an atom. □

Now assume by contradiction that 3∈L((−U)U). Then there are $T_{1},\;T_{2},\;T_{3}\in A\left(G\right)$ with $T_{1}T_{2}T_{3}=\left(-U\right)U$. We write

$$T_{j}=e_{1}^{k_{j}}{\left(-e_{1}\right)}^{k_{j}^{\prime }}\underset{i\in I_{j}}{\prod }\left(x_{i}e_{1}+e_{2}\right)\underset{i\in J_{j}}{\prod }\left(-x_{i}e_{1}-e_{2}\right)$$

for j∈[1,3]. Then $I_{1}⊎I_{2}⊎I_{3}=J_{1}⊎J_{2}⊎J_{3}=\left[1,n_{2}\right]$.

*Case* 1.1. Some $T_{i}$ has length 2, say w.l.o.g. $\left|T_{1}\right|=2$.

If $T_{2}$ or $T_{3}$ also has length 2, say w.l.o.g. $T_{2}$, then $\left|T_{3}\right|=\left|\left(-U\right)U\right|-\left|T_{1}\right|-\left|T_{2}\right|=2m+2mn-6>m+mn-1=D\left(G\right)$ is a contradiction. So $\left|T_{2}\right|,\left|T_{3}\right|>2$, and therefore $\left|\mathrm{supp}\left(T_{i}\right)\cap \left\{e_{1},-e_{1}\right\}\right|\leq 1$ for i∈[2,3]. After renumbering if necessary, we find

$$T_{2}=e_{1}^{n_{1}-k}\underset{i\in I_{2}}{\prod }\left(x_{i}e_{1}+e_{2}\right)\underset{i\in J_{2}}{\prod }\left(-x_{i}e_{1}-e_{2}\right)\;\text{and}$$

$$T_{3}={\left(-e_{1}\right)}^{n_{1}-k}\underset{i\in I_{3}}{\prod }\left(x_{i}e_{1}+e_{2}\right)\underset{i\in J_{3}}{\prod }\left(-x_{i}e_{1}-e_{2}\right)$$

with k∈{1,2} and k=2 only possible if $T_{1}=\left(-e_{1}\right)\left(e_{1}\right)$. Since $n_{1}\geq 4$ and k≤2, we have $n_{1}-k\geq 2$, whence $T_{2}\neq e_{1}\prod_{i=1}^{n_{2}}\left(x_{i}e_{1}+e_{2}\right)$ and $T_{3}\neq \left(-e_{1}\right)\prod_{i=1}^{n_{2}}\left(-x_{i}e_{1}-e_{2}\right)$. Consequently, Assertion A implies that $\left|I_{2}\right|=\left|J_{2}\right|\leq k$ and $\left|I_{3}\right|=\left|J_{3}\right|\leq k$. But now, if $T_{1}=\left(-e_{1}\right)\left(e_{1}\right)$, then $n_{2}=\left|I_{2}\right|+\left|I_{3}\right|\leq 2k=4$, a contradiction, while if instead $T_{1}=\left(x_{i}e_{1}+e_{2}\right)\left(-x_{i}e_{1}-e_{2}\right)$ for some $i\in \left[1,n_{2}\right]$, then $n_{2}-1=\left|I_{2}\right|+\left|I_{3}\right|\leq 2k=2$, also a contradiction.

*Case* 1.2. $\left|T_{i}\right|>2$ for all i∈[1,3].

Then $I_{i}\cap J_{i}=0̸$ and $\left|\mathrm{supp}\left(T_{i}\right)\cap \left\{e_{1},-e_{1}\right\}\right|\leq 1$ for all i∈[1,3], and thus by the pigeonhole principle, we find, after renumbering and possibly switching $e_{1}$ and $-e_{1}$ if necessary, that $e_{1}^{n_{1}-1}|T_{1}$. Thus, by A, it follows that $\left|I_{1}\right|=\left|J_{1}\right|=1$, say $I_{1}=\left\{a\right\}$ and $J_{1}=\left\{b\right\}$, and therefore $\left|I_{2}\right|+\left|I_{3}\right|=n_{2}-1$ and $\left|J_{2}\right|+\left|J_{3}\right|=n_{2}-1$.

Now, since $\left|I_{1}\right|,\;\left|J_{1}\right|\geq 1$ and since $\left|\mathrm{supp}\left(T_{i}\right)\cap \left\{e_{1},-e_{1}\right\}\right|\leq 1$ for all i∈[1,3], we see that we can again imply Assertion **A** to conclude $\left|I_{2}\right|=\left|J_{2}\right|$ and $\left|I_{3}\right|=\left|J_{3}\right|$.

Since $\left|T_{2}\right|,\;\left|T_{3}\right|>2$, it follows that $I_{2}\cap J_{2}=0̸$ and $I_{3}\cap J_{3}=0̸$. Thus we find that

$$I_{2}\subset J_{3}\cup \left\{b\right\}\text{,}\;I_{3}\subset J_{2}\cup \left\{b\right\}\text{,}\;J_{2}\subset I_{3}\cup \left\{a\right\}\;\text{and}\;J_{3}\subset I_{2}\cup \left\{a\right\}\text{.}$$

Consequently, if $I_{2}\cap J_{3}=0̸$, then $J_{3}\subset I_{2}\cup \left\{a\right\}$ would imply $\left|I_{3}\right|=\left|J_{3}\right|\leq 1$, in which case $I_{2}\subset J_{3}\cup \left\{b\right\}$ would further imply $\left|I_{2}\right|\leq \left|J_{3}\right|+1\leq 2$, and then $n_{2}-1=\left|I_{2}\right|+\left|I_{3}\right|\leq 2+1$ follows, contradicting that $n_{2}\geq 5$. Therefore there is some $\alpha \in I_{2}\cap J_{3}$. Similarly, if $I_{3}\cap J_{2}=0̸$, then $J_{2}\subset I_{3}\cup \left\{a\right\}$ would imply $\left|I_{2}\right|=\left|J_{2}\right|\leq 1$, whence $I_{3}\subset J_{2}\cup \left\{b\right\}$ would further imply $\left|I_{3}\right|\leq 2$, and then $n_{2}-1=\left|I_{2}\right|+\left|I_{3}\right|\leq 1+2$ follows, contradicting that $n_{2}\geq 5$. Therefore, we conclude that there is some $\beta \in I_{3}\cap J_{2}$.

But now, since there exists $\alpha \in I_{2}\cap J_{3}$ and $\beta \in I_{3}\cap J_{2}$, we have

$$\left(x_{\alpha }e_{1}+e_{2}\right)\left(-x_{\beta }e_{1}-e_{2}\right)|T_{2}\;\text{and}\;\left(-x_{\alpha }e_{1}-e_{2}\right)\left(x_{\beta }e_{1}+e_{2}\right)|T_{3}\text{.}$$

Let $k\in \left[0,n_{1}-1\right]$ be the integer such that ${\left(-e_{1}\right)}^{k}|T_{2}$ and ${\left(-e_{1}\right)}^{n_{1}-1-k}|T_{3}$ and let $l\in \left[0,n_{1}-1\right]$ be the integer such that $x_{\alpha }-x_{\beta }\equiv l\mathrm{mod}n_{1}$.

Suppose l≤k. Then

$${\left(-e_{1}\right)}^{l}\left(x_{\alpha }e_{1}+e_{2}\right)\left(-x_{\beta }e_{1}-e_{2}\right)|T_{2}$$

is either a proper zero-sum subsequence of $T_{2}$, contradicting that $T_{2}$ is an atom, or else $\left|I_{2}\right|=\left|J_{2}\right|=1$. However, in the latter case, we derive from $I_{3}\subset J_{2}\cup \left\{b\right\}$ that $\left|I_{3}\right|\leq \left|J_{2}\right|+1\leq 2$, whence $n_{2}=\left|I_{1}\right|+\left|I_{2}\right|+\left|I_{3}\right|\leq 1+1+2$, contradicting that $n_{2}\geq 5$. So we can instead assume $l\in \left[k+1,n_{1}-1\right]$.

In this case, $-l\equiv l^{\prime }\mathrm{mod}n_{1}$ for some $l^{\prime }\in \left[1,n_{1}-1-k\right]$, and thus we again find a contradiction by applying the same argument as above using $l^{\prime }$ and $T_{3}$ in place of l and $T_{2}$. This completes Case 1.

*Case* 2. U has type II in Theorem 3.1.

In this case, we have a basis $\left(e_{1},\;e_{2}\right)$ of G, with $\mathrm{ord}\left(e_{2}\right)=mn$ and $\mathrm{ord}\left(e_{1}\right)=m$, such that

$$U={\left(e_{1}+ye_{2}\right)}^{sm-1}e_{2}^{\left(n-s\right)m+\epsilon }\overset{m-\epsilon }{\underset{i=1}{\prod }}\left(-x_{i}e_{1}+\left(-x_{i}y+1\right)e_{2}\right)\text{,}$$

where $y\in \left[0,mn-1\right],\;\epsilon \in \left[1,m-1\right],\;s\in \left[1,n-1\right],\;x_{i}\in \left[1,m-1\right],\;\sum_{i=1}^{m-\epsilon }x_{i}=m-1$ and

(3.1) $$mye_{2}\neq 0\text{;}$$

furthermore,

(3.2) $$\text{either }s=1\;\text{or}\;mye_{2}=me_{2}\text{.}$$

Let $W=\prod_{i=1}^{m-\epsilon }\left(-x_{i}e_{1}+\left(-x_{i}y+1\right)e_{2}\right)$. Since s∈[1,n−1], we must have n≥2.

Assume by contradiction that we have a factorization $\left(-U\right)U=V_{1}V_{2}V_{3}$ with $V_{1},\;V_{2},\;V_{3}\in A\left(G\right)$. For each j∈[1,3], factor $V_{j}=S_{j}T_{j}$ such that $\mathrm{supp}\left(S_{j}\right)\subset \pm \left\{e_{1}+ye_{2},e_{2}\right\}$ and

$$T_{j}=\underset{i\in I_{j}}{\prod }\left(-x_{i}e_{1}+\left(-x_{i}y+1\right)e_{2}\right)\underset{i\in J_{j}}{\prod }\left(x_{i}e_{1}+\left(x_{i}y-1\right)e_{2}\right)\text{,}$$

where $I_{j},\;J_{j}\subset \left[1,m-\epsilon \right]$. Let $\Delta_{j}=\left|I_{j}\right|-\left|J_{j}\right|$ and let $\sigma_{j}=-\sum_{i\in I_{j}}x_{i}+\sum_{i\in J_{j}}x_{i}\in Z$, so that

$$\sigma \left(T_{j}\right)=\sigma_{j}e_{1}+\left(\sigma_{j}y+\Delta_{j}\right)e_{2}\text{.}$$

From the description of U, we trivially have

(3.3) $$\Delta_{j}\in \left[-\left(m-\epsilon \right),m-\epsilon \right]\subset \left[-\left(m-1\right),m-1\right]\;\text{and}\;\sigma_{j}\in \left[-\left(m-1\right),m-1\right]\text{,}$$

for each j∈[1,3].

We begin by handling the case when $\left|V_{i}\right|=2$ for some i∈[1,3].

*Case* 2.1. Suppose $\left|V_{i}\right|=2$ for some i∈[1,3], say $\left|V_{1}\right|=2$.

Since |(−U)U|−4=2mn+2m−6>mn+m−1=D(G), there can be at most one atom $V_{i}$ with $\left|V_{i}\right|=2$. Therefore $\left|V_{2}\right|,\left|V_{3}\right|>2$. For every element a of supp(U), we cannot have both a and −a in $V_{2}$ (or in $V_{3}$). Hence, since $V_{1}$ already contains an element and its negative, $V_{2}V_{3}$ consists of pairs a(−a), with each pair split evenly between $V_{2}$ and $V_{3}$. In other words, $V_{3}=-V_{2}$ and thus

(3.4) $$\left|V_{2}\right|=D\left(G\right)-1=nm+m-2\text{.}$$

Without loss of generality, we either have

$${\left(e_{1}+ye_{2}\right)}^{sm-2}{\left(-e_{2}\right)}^{\left(n-s\right)m+\epsilon -1}\left|V_{2}\;\text{or}\;{\left(e_{1}+ye_{2}\right)}^{sm-2}e_{2}^{\left(n-s\right)m+\epsilon -1}\right|V_{2}\text{.}$$

*Case* 2.1.1. Suppose ${\left(e_{1}+ye_{2}\right)}^{sm-2}{\left(-e_{2}\right)}^{\left(n-s\right)m+\epsilon -1}|V_{2}$.

Suppose 1<s<n. Then ${\left(e_{1}+ye_{2}\right)}^{m}{\left(-e_{2}\right)}^{m}|V_{2}$. Since s>1, (3.2) implies $mye_{2}=me_{2}$. But now $\sigma \left({\left(e_{1}+ye_{2}\right)}^{m}{\left(-e_{2}\right)}^{m}\right)=me_{1}+mye_{2}-me_{2}=0$. As a result, since $V_{2}$ is an atom, we conclude that ${\left(e_{1}+ye_{2}\right)}^{m}{\left(-e_{2}\right)}^{m}=V_{2}$, so that (3.4) implies $2m=\left|V_{2}\right|=nm+m-2\geq 2m+m-2>2m$, a contradiction. So we instead conclude that s=1 (in view of s∈[1,n−1]).

If there is an $i\in I_{2}$ such that $x_{i}\leq m-2$, then ${\left(e_{1}+ye_{2}\right)}^{x_{i}}\left(-e_{2}\right)\left(-x_{i}e_{1}+\left(-x_{i}y+1\right)e_{2}\right)$ is a zero-sum subsequence of the atom $V_{2}$, whence $V_{2}={\left(e_{1}+ye_{2}\right)}^{x_{i}}\left(-e_{2}\right)\left(-x_{i}e_{1}+\left(-x_{i}y+1\right)e_{2}\right)$. But in such case, (3.4) yields $m-2+2\geq x_{i}+2=\left|V_{2}\right|=nm+m-2>m$, a contradiction. Therefore any $i\in I_{2}$ has $x_{i}=m-1$.

Thus, if $I_{2}$ is nonempty, then this is only possible, in view of $\sum_{i=1}^{m-\epsilon }x_{i}=m-1$ with $x_{i}\in \left[1,m-1\right]$, if ϵ=m−1,|W|=1 and $J_{2}=0̸$. So, recalling that $\left|V_{1}\right|=2$ and s=1, we necessarily find, in this case, that $V_{2}$ has the form

$$V_{2}={\left(e_{1}+ye_{2}\right)}^{m-2}{\left(-e_{2}\right)}^{\left(n-1\right)m+m-1}\left(e_{1}+\left(-my+y+1\right)e_{2}\right)\;\text{or}$$

$$V_{2}={\left(e_{1}+ye_{2}\right)}^{m-1}{\left(-e_{2}\right)}^{\left(n-1\right)m+m-2}\left(e_{1}+\left(-my+y+1\right)e_{2}\right)\text{.}$$

However, in the former case, $\sigma \left(V_{2}\right)$ has $e_{1}$-coordinate equal to $\left(m-1\right)e_{1}\neq 0$, while in the latter case, $\sigma \left(V_{2}\right)$ has $e_{2}$-coordinate equal to $3e_{2}\neq 0$ (in view of mn≥4). Since $V_{2}$ is zero-sum, these are both contradictions, and we thereby conclude that $I_{2}=0̸$.

If $V_{1}$ does not consist of a pair of terms from W, then $V_{2}$ must, in view of $I_{2}=0̸$ and (3.4), contain every term from $\prod_{i=1}^{m-\epsilon }\left(x_{i}e_{1}+\left(x_{i}y-1\right)e_{2}\right)$, i.e.,  $J_{2}=\left[1,m-\epsilon \right]$. But in this case, $\sigma \left(V_{2}\right)$ has $e_{1}$-coordinate equal to either $\left(m-2+\sum_{i=1}^{m-\epsilon }x_{i}\right)e_{1}=-3e_{1}$ or $\left(m-1+\sum_{i=1}^{m-\epsilon }x_{i}\right)e_{1}=-2e_{1}$, both nonzero in view of m≥4, thus contradicting that $V_{2}$ is zero-sum. Therefore, this only leaves the possibility of $V_{1}$ consisting of a pair of terms from W, in which case

$$V_{2}={\left(e_{1}+ye_{2}\right)}^{m-1}{\left(-e_{2}\right)}^{\left(n-1\right)m+\epsilon }\underset{i\in J_{2}}{\prod }\left(x_{i}e_{1}+\left(x_{i}y-1\right)e_{2}\right)$$

with $\left|J_{2}\right|=m-\epsilon -1$. Considering the $e_{1}$-coordinate of $\sigma \left(V_{2}\right)$, which must be zero, we conclude that $\sum_{i\in J_{2}}x_{i}\equiv 1\mathrm{mod}m$, which, in view of $\sum_{i\in J_{2}}x_{i}\in \left[0,m-1\right]$, implies $\sum_{i\in J_{2}}x_{i}=1$. Consequently, in view of $\left|J_{2}\right|=m-\epsilon -1$ and $x_{i}\in \left[1,m-1\right]$, it follows that $1=\left|J_{2}\right|=m-\epsilon -1$ with $x_{i}=1$ for the unique $i\in J_{2}$. But now the $e_{2}$-coordinate of $\sigma \left(V_{2}\right)$ is easily calculated to be $\left(\left(m-1\right)y+m-\epsilon +y-1\right)e_{2}=\left(my+1\right)e_{2}$. Since this must be zero with $\mathrm{ord}\left(e_{2}\right)=nm$, we must have my+1≡0modm, a subcase concluding contradiction.

*Case* 2.1.2. Suppose ${\left(e_{1}+ye_{2}\right)}^{sm-2}e_{2}^{\left(n-s\right)m+\epsilon -1}|V_{2}$

In this case, $J_{2}\neq 0̸$, for otherwise $V_{2}|U$, a contradiction.

Suppose $x_{i}>1$ for some $i\in J_{2}$. Then, in view of $\sum_{i=1}^{m-\epsilon }x_{i}=m-1$ with $x_{i}\in \left[1,m-1\right]$, we conclude that ϵ>1, whence

$$S={\left(e_{1}+ye_{2}\right)}^{sm-x_{i}}e_{2}^{\left(n-s\right)m+1}\left(x_{i}e_{1}+\left(x_{i}y-1\right)e_{2}\right)$$

is a subsequence of $V_{2}$. We claim that S now contains a nontrivial zero-sum subsequence. Indeed, if s>1, then (3.2) implies that $mye_{2}=me_{2}$, in which case a short calculation shows that S is itself a zero-sum sequence. On the other hand, if s=1, then $mye_{2}=-bme_{2}$ for some b∈[1,n−1] (in view of (3.1)), and now ${\left(e_{1}+ye_{2}\right)}^{m-x_{i}}e_{2}^{bm+1}\left(x_{i}e_{1}+\left(x_{i}y-1\right)e_{2}\right)$ is a nontrivial zero-sum subsequence of S, as claimed. Consequently, since S divides the atom $V_{2}$ and contains a nontrivial zero-sum subsequence, we conclude that $S=V_{2}$. Thus (3.4), $x_{i}\geq 2$ and m≥3 combine to imply $nm+m-2=\left|V_{2}\right|=\left|S\right|=nm+2-x_{i}\leq nm$, a contradiction. So we instead conclude that $x_{i}=1$ for every $i\in J_{2}$. In particular, since $J_{2}\neq 0̸$, we conclude that $\left(e_{1}+\left(y-1\right)e_{2}\right)$ is a term of $V_{2}$.

As a result, if $I_{2}\neq 0̸$, then ${\left(e_{1}+ye_{2}\right)}^{x_{i}-1}\left(-x_{i}e_{1}+\left(-x_{i}y+1\right)e_{2}\right)\left(e_{1}+\left(y-1\right)e_{2}\right)$ is a zero-sum subsequence of $V_{2}$ for any $i\in I_{2}$, and therefore must be equal to the atom $V_{2}$, whence (3.4) yields $mn+m-2=\left|V_{2}\right|=x_{i}+1\leq m$, contradicting m≥4. Therefore, we see that $I_{2}=0̸$. Consequently

$$V_{2}={\left(e_{1}+ye_{2}\right)}^{a_{1}}e_{2}^{a_{2}}{\left(e_{1}+\left(y-1\right)e_{2}\right)}^{a_{3}}$$

where $sm-2\leq a_{1}\leq sm-1,\;\left(n-s\right)m+\epsilon -1\leq a_{2}\leq \left(n-s\right)m+\epsilon ,\;\max\left\{1,m-\epsilon -1\right\}\leq a_{3}\leq m-\epsilon$, and exactly one of the $a_{i}$ does not achieve its upper bound. Since the $e_{1}$-coordinate of $\sigma \left(V_{2}\right)$ must be zero, it follows that $a_{1}+a_{3}\equiv 0\mathrm{mod}m$, which means that either $a_{1}=sm-2$ and $a_{3}=2$, or else $a_{1}=sm-1$ and $a_{3}=1$.

Suppose $a_{1}=sm-2$ and $a_{3}=2$. Then equality must hold in the upper bound for $a_{3}$, whence $2=a_{3}=m-\epsilon$ and $J_{2}=\left[1,m-\epsilon \right]$. In view of $J_{2}=\left[1,m-\epsilon \right]$ with $x_{i}=1$ for all $i\in J_{2}$, we obtain $m-1=\sum_{i=1}^{m-\epsilon }x_{i}=\sum_{i\in J_{2}}x_{i}=\left|J_{2}\right|=m-\epsilon =2$, where the final equality follows in view of $2=a_{3}=m-\epsilon$, contradicting that m≥4. So it remains to consider when $a_{1}=sm-1$ and $a_{3}=1$.

In this case, we either have $1=a_{3}=m-\epsilon$ and $a_{2}=\left(n-s\right)m+\epsilon -1$, or else $1=a_{3}=m-\epsilon -1$ and $a_{2}=\left(n-s\right)m+\epsilon$. In both cases, the $e_{2}$-coordinate of $\sigma \left(V_{2}\right)$ is $\left(sym-sm+m-3\right)e_{2}$. Thus, since $\mathrm{ord}\left(e_{2}\right)=mn$, we obtain the case concluding contradiction sym−sm+m−3≡0modm in view of m≥4.

*Case* 2.2. $\left|V_{i}\right|\geq 3$ for all i∈[1,3].

In this case, we conclude that the atoms $V_{j}$ cannot contain both terms equal to $e_{2}$ and $-e_{2}$. As a result, the pigeonhole principle guarantees that some $V_{j}$, say $V_{1}$, either contains all terms equal to $e_{2}$ or all terms equal to $-e_{2}$. Hence, by symmetry, we can w.l.o.g. assume

$$e_{2}^{\left(n-s\right)m+\epsilon }|V_{1}\text{.}$$

Suppose $\pm \left(e_{1}+ye_{2}\right)\notin \mathrm{supp}\left(V_{1}\right)$. Then, by considering the $e_{1}$-coordinate of $\sigma \left(V_{1}\right)$, we conclude that $\sigma_{1}=0$. In particular, we cannot have $\left|I_{1}\right|=m-\epsilon$ or $\left|J_{1}\right|=m-\epsilon$, since that would force $\pm \sigma_{1}=\pm \sum_{i=1}^{m-\epsilon }x_{i}=\pm \left(m-1\right)\neq 0$. Consequently, we have

(3.5) $$\sigma \left(T_{1}\right)=\Delta_{1}e_{2}\in \left[-\left(m-\epsilon -1\right),m-\epsilon -1\right]\cdot e_{2}\text{.}$$

However, since $-\sigma \left(T_{1}\right)=\sigma \left(V_{1}T_{1}^{-1}\right)=\left|V_{1}T_{1}^{-1}\right|e_{2}=\left(\left(n-s\right)m+\epsilon \right)e_{2}$, it follows that the $e_{2}$-coordinate of $\sigma \left(T_{1}\right)$ is congruent to sm−ϵ modulo mn. However, since sm−ϵ≥m−ϵ and −nm+sm−ϵ≤−(m−ϵ), we see that the $e_{2}$-coordinate of $\sigma \left(T_{1}\right)$ being congruent to sm−ϵ modulo mn is contrary to (3.5). So we instead conclude that $\left(e_{1}+ye_{2}\right)\in \mathrm{supp}\left(V_{1}\right)$ or $\left(-e_{1}-ye_{2}\right)\in \mathrm{supp}\left(V_{1}\right)$, which gives us two subcases

*Case* 2.2.1. $v_{-e_{1}-ye_{2}}\left(V_{1}\right)>0$.

Let $v_{-e_{1}-ye_{2}}\left(V_{1}\right)=s^{\prime }m+l>0$, where $s^{\prime }\in \left[0,s-1\right]$ and l∈[0,m−1]. Considering the sum of the $e_{1}$-coordinates of the terms of $V_{1}$, we conclude that $\sigma_{1}\equiv l\mathrm{mod}m$. Thus, in view of (3.3), we have $\sigma_{1}\in \left\{l,l-m\right\}$. But now, considering the sum of the $e_{2}$-coordinates of the terms of $V_{1}$ modulo m, we conclude that $\Delta_{1}\equiv -\epsilon \mathrm{mod}m$, which in view of (3.3) forces $\Delta_{1}\in \left\{m-\epsilon ,-\epsilon \right\}$.

Suppose $\Delta_{1}=-\epsilon <0$. Then there will be at least ϵ terms from −W contained in $V_{1}$. If one of these terms is equal to $e_{1}+\left(y-1\right)e_{2}$, then

$$\left(-e_{1}-ye_{2}\right)\left(e_{1}+\left(y-1\right)e_{2}\right)e_{2}$$

will be a proper zero-sum subsequence of $V_{1}$, contradicting that $V_{1}$ is an atom. Therefore we instead have $x_{i}\geq 2$ for each of the ϵ terms of $V_{1}$ from −W. Since the remaining m−2ϵ terms of −W have $x_{i}\geq 1$, we obtain the estimate $m-1=\sum_{i=1}^{m-\epsilon }x_{i}\geq 2\epsilon +m-2\epsilon =m$, which is a contradiction. So we cannot have $\Delta_{1}=-\epsilon$, and instead conclude that $\Delta_{1}=m-\epsilon$.

However, $\Delta_{1}=m-\epsilon$ is only possible if $V_{1}$ contains all m−ϵ terms of W and no term from −W, in which case $\sigma_{1}=-\left(m-1\right)\in \left\{l,l-m\right\}$. Hence $\sigma_{1}=l-m$ with l=1. But now

(3.6) $$0=\sigma \left(V_{1}\right)=\left(-s^{\prime }my-ly+\sigma_{1}y+\Delta_{1}+\left(n-s\right)m+\epsilon \right)e_{2}=-\left(\left(s^{\prime }+1\right)my+\left(s-1\right)m\right)e_{2}\text{.}$$

If s=1, then $s^{\prime }\in \left[0,s-1\right]$ forces $s^{\prime }=0$, whence (3.6) implies $mye_{2}=0$, contrary to (3.1). Therefore we can assume s≥2, in which case (3.2) implies $mye_{2}=me_{2}$. But then

$$W\left(-e_{1}-ye_{2}\right)e_{2}^{\epsilon }$$

is a proper zero-sum subsequence of $V_{1}$, contradicting that $V_{1}$ is an atom. This completes Case 2.2.1.

*Case* 2.2.2. $v_{e_{1}+ye_{2}}\left(V_{1}\right)>0$.

Let $v_{e_{1}+ye_{2}}\left(V_{1}\right)=s^{\prime }m+l>0$, where $s^{\prime }\in \left[0,s-1\right]$ and l∈[0,m−1]. Considering the sum of the $e_{1}$-coordinates of the terms of $V_{1}$, we conclude that $\sigma_{1}\equiv -l\mathrm{mod}m$. Thus, in view of (3.3), we have $\sigma_{1}\in \left\{-l,m-l\right\}$. But now, considering the sum of the $e_{2}$-coordinates of the terms of $V_{1}$ modulo m, we conclude that $\Delta_{1}\equiv -\epsilon \mathrm{mod}m$, which in view of (3.3) forces $\Delta_{1}\in \left\{m-\epsilon ,-\epsilon \right\}$. Since $\Delta_{1}=m-\epsilon$ is only possible if $V_{1}$ contains all m−ϵ terms of W and none from −W, we see that $\Delta_{1}=m-\epsilon$ would imply $V_{1}|U$, which is not possible as U has no proper nontrivial zero-sum subsequences. Therefore

$$\Delta_{1}=-\epsilon \text{.}$$

Thus

(3.7) $$0=\sigma \left(V_{1}\right)=\left(s^{\prime }my+ly+\sigma_{1}y-sm\right)e_{2}\text{.}$$

Suppose $\sigma_{1}=-l$. If also s=1, then $s^{\prime }=0$, whence (3.7) implies $me_{2}=0$, contradicting that $\mathrm{ord}\left(e_{2}\right)=mn\geq 2m$. On the other hand, if s>1, then $mye_{2}=me_{2}$, whence (3.7) instead implies $\left(s^{\prime }-s\right)me_{2}=0$. Thus $s^{\prime }\equiv s\mathrm{mod}n$. However, since $s^{\prime }\in \left[0,s-1\right]\subset \left[0,n-2\right]$, this is not possible. So we conclude that $\sigma_{1}=-l$ is not possible, and we must instead have

$$\sigma_{1}=m-l\text{.}$$

If s>1, then (3.2) implies that

(3.8) $$mye_{2}=me_{2}$$

holds. On the other hand, if s=1, then $s^{\prime }=0$, whence (3.7) yields $\left(my-m\right)e_{2}=0$, and now (3.8) holds again. Thus we now know (3.8) holds in all cases.

From (3.7), (3.8), we derive that $\left(s^{\prime }-s+1\right)me_{2}=0$, whence $s^{\prime }\equiv s-1\mathrm{mod}n$. Thus, since $s^{\prime }\in \left[0,s-1\right]\subset \left[0,n-2\right]$, we conclude that

$$s^{\prime }=s-1\text{.}$$

Also, since $m-l=\sigma_{1}\leq m-1$ holds by (3.3), we conclude that l≠0, and thus l∈[1,m−1].

As in Case 2.2.1, if all terms of $V_{1}$ from −W have $x_{i}\geq 2$, then we obtain the contradiction $m-1=\sum_{i=1}^{m-\epsilon }x_{i}\geq 2\epsilon +m-2\epsilon =m$. Therefore there must be some term of $V_{1}$ equal to $e_{1}+\left(y-1\right)e_{2}$, i.e., 

(3.9) $$v_{e_{1}+\left(y-1\right)e_{2}}\left(V_{1}\right)>0\text{.}$$

Suppose l=m−1. Then

$${\left(e_{1}+ye_{2}\right)}^{sm-1}\left(e_{1}+\left(y-1\right)e_{2}\right)e_{2}^{\left(n-s\right)m+1}$$

will be a zero-sum subsequence of $V_{1}$ in view of (3.8). Moreover, it will be proper, contradicting that $V_{1}$ is an atom, unless ϵ=1 and $V_{1}$ contains only one term from W(−W), which must be equal to $e_{1}+\left(y-1\right)e_{2}$. However, ϵ=1 together with $\sum_{i=1}^{m-\epsilon }x_{i}=m-1$ and $x_{i}\geq 1$ then forces $x_{i}=1$ for all i∈[1,m−1]. But now the only terms of U not contained in $V_{1}$ are all equal to $-e_{1}+\left(-y+1\right)e_{2}$. Since each atom $V_{j}$ must contain a term from U and a term from −U, we see that $-e_{1}+\left(-y+1\right)e_{2}\in \mathrm{supp}\left(V_{2}\right)\cap \mathrm{supp}\left(V_{3}\right)$. However, $V_{2}$ and $V_{3}$ must also contain the remaining m−2≥2 terms of −W all equal to $e_{1}+\left(y-1\right)e_{2}$, which forces $V_{2}=V_{3}=\left(-e_{1}+\left(-y+1\right)e_{2}\right)\left(e_{1}+\left(y-1\right)e_{2}\right)$, contradicting that $V_{1}V_{2}V_{3}=\left(-U\right)U$. So we instead conclude that

l∈[1,m−2].

Since l<m−1 and $\left|V_{i}\right|\geq 3$ for all i∈[1,3], we see that one of either $V_{2}$ or $V_{3}$, say $V_{3}$, must contain all remaining m−1−l>0 terms equal to $e_{1}+ye_{2}$, while the other atom $V_{2}$ must contain all sm−1 terms equal to $-e_{1}-ye_{2}$. In summary,

(3.10) $$v_{e_{1}+ye_{2}}\left(V_{3}\right)=m-1-l\;\text{and}\;v_{-e_{1}-ye_{2}}\left(V_{2}\right)=sm-1\text{.}$$

Let us next examine the atom $V_{2}$ more closely. Letting β∈[0,(n−s)m+ϵ] be the multiplicity of $-e_{2}$ in $V_{3}$, we derive that (n−s)m+ϵ−β is the multiplicity of $-e_{2}$ in $V_{2}$. If β=0, then

$${\left(-e_{1}-ye_{2}\right)}^{sm-1}{\left(-e_{2}\right)}^{\left(n-s\right)m+\epsilon }|V_{2}\text{,}$$

in which case, by symmetry, we are in the same situation as when l=m−1 for the atom $V_{1}$ (simply swap $e_{2}$ for $-e_{2}$ in the arguments) and obtain the corresponding contradiction. Therefore

β≥1.

In view of (3.10), we see by summing the $e_{1}$-coordinates of the terms of $V_{2}$ that $\sigma_{2}\equiv -1\mathrm{mod}m$. Thus, in view of (3.3), we have $\sigma_{2}\in \left\{-1,m-1\right\}$, However, if $\sigma_{2}=m-1$, then $V_{2}$ must contain all terms from −W, which is not possible since we showed earlier that $V_{1}$ contains a term from −W equal to $e_{1}+\left(y-1\right)e_{2}$ (see (3.9)). Therefore we conclude that

$$\sigma_{2}=-1\text{.}$$

But then

$$0=\sigma \left(V_{2}\right)=\left(-\left(sm-1\right)y-y+\Delta_{2}+sm-\epsilon +\beta \right)e_{2}\text{.}$$

In view of the above equation and (3.8), we derive

$$\Delta_{2}\equiv \epsilon -\beta \mathrm{mod}mn\text{.}$$

As a result, observing that nm+ϵ−β≥nm+ϵ−((n−s)m+ϵ)≥m and that −mn+ϵ−β≤−mn+m−1≤−m−1, we conclude from (3.3) that

$$\Delta_{2}=\epsilon -\beta \in \left[-\left(m-\epsilon \right),m-\epsilon \right]\text{.}$$

However, we can slightly improve this estimate by recalling that $\Delta_{1}=-\epsilon$ forced there to be at least ϵ terms of $V_{1}$ from −W, leaving only at most m−2ϵ terms from −W available for $V_{2}$. Thus

(3.11) $$\Delta_{2}=\epsilon -\beta \in \left[-\left(m-2\epsilon \right),m-\epsilon \right]\text{.}$$

From (3.11), we infer that

(3.12) β≤m−ϵ.

Let us next examine the atom $V_{3}$ more closely. Noting that $\sigma_{1}+\sigma_{2}+\sigma_{3}=0$, we deduce that

$$\sigma_{3}=-\left(\sigma_{1}+\sigma_{2}\right)=l+1-m\text{.}$$

Noting that $\Delta_{1}+\Delta_{2}+\Delta_{3}=0$, we deduce that

$$\Delta_{3}=-\Delta_{1}-\Delta_{2}=\epsilon -\epsilon +\beta =\beta \text{.}$$

Since $\Delta_{3}=\beta \geq 1$, we see that there must be at least β terms of $V_{3}$ from W.

In view of (3.9) and $\left|V_{i}\right|\geq 3$ for all i∈[1,3], we find that the term $-e_{1}+\left(-y+1\right)e_{2}$ must be contained in either $V_{2}$ or $V_{3}$. This gives 2 final subcases.

*Case* 2.2.2.1. Suppose $-e_{1}+\left(-y+1\right)e_{2}\notin \mathrm{supp}\left(V_{3}\right)$.

Then $-e_{1}+\left(-y+1\right)e_{2}\in \mathrm{supp}\left(V_{2}\right)$ and all of the at least β terms of $V_{3}$ from W have $x_{i}\geq 2$. Thus we obtain the estimate

$$2\beta +\left(m-\epsilon -\beta \right)\leq \overset{m-\epsilon }{\underset{i=1}{\sum }}x_{i}=m-1\text{,}$$

yielding β≤ϵ−1. Since $-e_{1}+\left(-y+1\right)e_{2}\in \mathrm{supp}\left(V_{2}\right)$ and β≤ϵ−1, we see in view of (3.8) that

$${\left(-e_{2}\right)}^{\left(n-s\right)m+1}{\left(-e_{1}-ye_{2}\right)}^{sm-1}\left(-e_{1}+\left(-y+1\right)e_{2}\right)$$

is a zero-sum subsequence of $V_{2}$. Thus we contradict that $V_{2}$ is an atom unless β=ϵ−1 and $\left(-e_{1}+\left(-y+1\right)e_{2}\right)$ is the unique term of $V_{2}$ from W(−W). However, equality in the estimate β≤ϵ−1 is only possible if $V_{3}$ contains exactly β terms from W, which in view of $\Delta_{3}=\beta$ is only possible if $V_{3}$ contains no terms of −W. Furthermore, each of the β terms of $V_{3}$ from W must have $x_{i}=2$—else we again contradict that equality holds in the estimate β≤ϵ−1. In particular, since β≥1, we see that

(3.13) $$\left(-2e_{1}+\left(-2y+1\right)e_{2}\right)\in \mathrm{supp}\left(V_{3}\right)\text{.}$$

Since we have just derived that neither $V_{2}$ nor $V_{3}$ contains terms from −W, it follows that $V_{1}$ contains all the terms from −W, and thus none from W in view of $\left|V_{1}\right|\geq 3$. Consequently, it follows that $m-l=\sigma_{1}=m-1$, implying l=1. However, in view of β≥1, (3.13), and l=1 with m≥5, it follows that

$${\left(e_{1}+ye_{2}\right)}^{2}\left(-e_{2}\right)\left(-2e_{1}+\left(-2y+1\right)e_{2}\right)$$

is a proper zero-sum subsequence of $V_{3}$, contradicting that $V_{3}$ is an atom.

*Case* 2.2.2.2. Suppose $-e_{1}+\left(-y+1\right)e_{2}\in \mathrm{supp}\left(V_{3}\right)$.

Since m−l−1≥1,β≥1 and $-e_{1}+\left(-y+1\right)e_{2}\in \mathrm{supp}\left(V_{3}\right)$, we find that

$$\left(e_{1}+ye_{2}\right)\left(-e_{1}+\left(-y+1\right)e_{2}\right)\left(-e_{2}\right)$$

is a zero-sum subsequence of $V_{3}$. Since $V_{3}$ is an atom, this cannot be a proper subsequence, which implies

$$l=m-2\text{,}\;\beta =1\text{,}\;\text{and that }-e_{1}+\left(-y+1\right)e_{2}\text{ is the only term of }V_{3}\text{ from }W\left(-W\right)\text{.}$$

Since $\Delta_{1}=-\epsilon <0$, we know that there are ϵ terms of $V_{1}$ from −W. However, if these were all the terms of −W, then $V_{1}$ could contain no terms from W (in view of $\left|V_{1}\right|\geq 3$) and we would have $m-l=\sigma_{1}=m-1$; hence l=1, contradicting that l=m−2 with m≥4. As a result, we see that the ϵ terms of $V_{1}$ from −W cannot be all the terms of −W, from which we derive that ϵ<|W|=m−ϵ, and thus that

$$\epsilon <\frac{m}{2}\text{.}$$

We established in (3.9) that $x_{i}=1$ for some i∈[1,m−ϵ]. However, if there is only one i∈[1,m−ϵ] such that $x_{i}=1$, then we would obtain the estimate $m-1=\sum_{i=1}^{m-\epsilon }x_{i}\geq 2\left(m-\epsilon -1\right)+1$, contradicting that $\epsilon <\frac{m}{2}$. As a result, we see that ${\left(-e_{1}+\left(-y+1\right)e_{2}\right)}^{2}|U\left(-U\right)$. In view of (3.9) and $\left|V_{1}\right|\geq 3$, we see that $V_{1}$ cannot contain a term equal to $-e_{1}+\left(-y+1\right)e_{2}$. On the other hand, since $-e_{1}+\left(-y+1\right)e_{2}$ is the only term of $V_{3}$ from W(−W), we see that $V_{3}$ cannot contain both terms of U(−U) equal to $-e_{1}+\left(-y+1\right)e_{2}$. In consequence, we conclude that $-e_{1}+\left(-y+1\right)e_{2}\in \mathrm{supp}\left(V_{2}\right)$.

But now, if ϵ≥2, then

$${\left(-e_{2}\right)}^{\left(n-s\right)m+1}{\left(-e_{1}-ye_{2}\right)}^{sm-1}\left(-e_{1}+\left(-y+1\right)e_{2}\right)$$

is a zero-sum subsequence of $V_{2}$ in view of (3.8). Furthermore, it will be a proper zero-sum subsequence, contradicting that $V_{2}$ is an atom, unless ϵ=2 and $\left(-e_{1}+\left(-y+1\right)e_{2}\right)$ is the only term of $V_{2}$ from W(−W). However, in such case, we would have $\left|T_{2}\right|+\left|T_{3}\right|=2$, which is only possible, in view of $\left|V_{1}\right|\geq 3$, if the 2 terms of $T_{2}$ and $T_{3}$, both with $x_{i}=1$, cover all m−ϵ terms of W. But this implies $2=\sum_{i=1}^{m-\epsilon }x_{i}=m-1$, contradicting that m≥4. Thus it remains only to consider the case when ϵ=1, which, in view of $\sum_{i=1}^{m-\epsilon }x_{i}=m-1$ with $x_{i}\in \left[1,m-1\right]$, is only possible if $x_{i}=1$ for all i∈[1,m−ϵ]=[1,m−1].

Since ϵ=1 and β=1, we have $\Delta_{2}=\epsilon -\beta =0$. In consequence, we see that $V_{2}$ must contain an equal number of terms from W and from −W. However, since $x_{i}=1$ for all i∈[1,m−ϵ] and since $\left|V_{2}\right|\geq 3$, this forces $V_{2}$ to contain no terms from W(−W) at all, whence $\sigma_{2}=0$, contradicting that we already showed $\sigma_{2}=-1$, thus completing the proof. □

Theorem 3.5 *Let* H *be a Krull monoid with class group* $G≅C_{m}⊕C_{mn}$ *, where* m,n∈N *and* m≥2 *, and suppose that every class contains a prime divisor. Then*

$$U_{\left\{2,D\left(G\right)\right\}}\left(H\right)=\left\{ \begin{array}{cc} \left\{2a|a\in \left[1,n\right]\right\}\cup \left\{D\left(G\right)\right\} & m=2\text{,}\\ \left[2,D\left(G\right)\right] & m\in \left[3,4\right]\text{,}\\ \left[2,D\left(G\right)\right]\setminus \left\{3\right\} & m\geq 5\text{.} \end{array}\right.$$

Proof By Lemma 2.1, it suffices to prove the assertion for the monoid B(G) where $G=C_{m}⊕C_{mn}$. We have $D\left(G\right)=D^{*}\left(G\right)=m+mn-1$ and set M={2,D(G)}. Recall that

$$\left\{2,m\right\}\cup \left[mn,D\left(G\right)\right]\subset U_{M}\left(G\right)\subset \left[2,D\left(G\right)\right]$$

by Lemmas 3.2.1 and 3.3 (with $G_{1}=\left\{0\right\}$). Moreover, by Proposition 3.4, we have $3\notin U_{M}\left(G\right)$ if m≥5. We choose a basis $\left(e_{1},e_{2}\right)$ of G with $\mathrm{ord}\left(e_{1}\right)=m$ and $\mathrm{ord}\left(e_{2}\right)=mn$ and provide a series of examples which cover all cases.

*Case* 1. m=2. This follows from Lemma 3.2.2.

*Case* 2. m=3. For j∈[1,3n−1], we set

$$U=e_{2}^{3n-1}e_{1}\left(e_{1}+je_{2}\right)\left(e_{1}+\left(1-j\right)e_{2}\right)\text{,}$$

$$V_{1}=e_{2}^{3n-j}\left(e_{1}+je_{2}\right)\left(-e_{1}\right)\text{,}$$

$$V_{2}=\left(e_{1}+\left(1-j\right)e_{2}\right)\left(-e_{1}+\left(j-1\right)e_{2}\right)\text{,}$$

$$V_{3}=e_{2}\left(-e_{2}\right)\text{.}$$

We have $U,V_{1},V_{2},V_{3}\in A\left(G\right),\left|U\right|=D\left(G\right),V_{1}\left(-V_{1}\right)V_{2}V_{3}^{j-1}=\left(-U\right)U$, and thus 2+j∈L((−U)U) for j∈[1,3n−1]. This shows $\left[3,3n+1\right]\subset U_{M}\left(G\right)$.

*Case* 3. m=4. For j∈[2,4n−2], we set

$$U=e_{2}^{4n-1}e_{1}\left(e_{1}+e_{2}\right)\left(e_{1}+je_{2}\right)\left(e_{1}-je_{2}\right)\text{,}$$

$$V_{1}=e_{2}^{j+1}\left(e_{1}-je_{2}\right)\left(-e_{1}-e_{2}\right)\text{,}$$

$$V_{2}={\left(-e_{2}\right)}^{j}\left(e_{1}+je_{2}\right)\left(-e_{1}\right)\text{,}$$

$$V_{3}=\left(-e_{2}\right)e_{1}\left(e_{1}+e_{2}\right)\left(-e_{1}-je_{2}\right)\left(-e_{1}+je_{2}\right)\text{,}$$

$$V_{4}=e_{2}\left(-e_{2}\right)\text{.}$$

We have $U,V_{1},\ldots ,V_{4}\in A\left(G\right),\left|U\right|=D\left(G\right)$, $V_{1}V_{2}V_{3}V_{4}^{4n-2-j}=\left(-U\right)U$, and thus 4n+1−j∈L((−U)U) for j∈[2,4n−2]. This shows $\left[3,4n-1\right]\subset U_{M}\left(G\right)$.

*Case* 4. m≥5 odd. We begin by showing that $4\in U_{M}\left(G\right)$. Set

$$U=e_{2}^{mn-1}{\left(ne_{2}+e_{1}\right)}^{\frac{m-1}{2}-1}\left(-2ne_{2}+e_{1}\right){\left(-ne_{2}+e_{1}\right)}^{\frac{m-1}{2}-1}\left(\left(2n+1\right)e_{2}+e_{1}\right)e_{1}\text{,}$$

$$V_{1}=e_{2}^{mn-n}\left(ne_{2}+e_{1}\right)\left(-e_{1}\right)\text{,}$$

$$V_{2}={\left(-e_{2}\right)}^{mn-n}\left(-2ne_{2}+e_{1}\right)\left(ne_{2}-e_{1}\right)\text{,}$$

$$V_{3}={\left(ne_{2}+e_{1}\right)}^{\frac{m-1}{2}-2}{\left(ne_{2}-e_{1}\right)}^{\frac{m-1}{2}-2}\left(\left(2n+1\right)e_{2}+e_{1}\right)\left(2ne_{2}-e_{1}\right)e_{2}^{n-1}\text{,}$$

$$V_{4}={\left(-ne_{2}+e_{1}\right)}^{\frac{m-1}{2}-1}{\left(-ne_{2}-e_{1}\right)}^{\frac{m-1}{2}-1}\left(-\left(2n+1\right)e_{2}-e_{1}\right)e_{1}{\left(-e_{2}\right)}^{n-1}\text{.}$$

We have $U,\;V_{1},\ldots ,V_{4}\in A\left(G\right),\left|U\right|=D\left(G\right),V_{1}V_{2}V_{3}V_{4}=\left(-U\right)U$, and thus $4\in L\left(\left(-U\right)U\right)\subset U_{M}\left(G\right)$. It remains to show $\left[5,mn-1\right]\subset U_{M}\left(G\right)$.

For i∈[0,mn−1], we set

$$U=e_{2}^{mn-1}{\left(e_{2}+e_{1}\right)}^{\frac{m-5}{2}}\left(\left(m+i+1\right)e_{2}+e_{1}\right)\left(\left(3-m\right)e_{2}+e_{1}\right){\left(-e_{2}+e_{1}\right)}^{\frac{m-5}{2}}\left(\left(-1-i\right)e_{2}+e_{1}\right)\left(-2e_{2}+e_{1}\right)e_{1}\text{,}$$

$$V_{1}={\left(e_{2}+e_{1}\right)}^{\frac{m-5}{2}}\left(\left(3-m\right)e_{2}+e_{1}\right){\left(e_{2}-e_{1}\right)}^{\frac{m-5}{2}}\left(2e_{2}-e_{1}\right)\text{,}$$

$$V_{2}=e_{2}^{mn-1-i}e_{1}\left(\left(1+i\right)e_{2}-e_{1}\right)\text{,}$$

$$V_{3}=\left(\left(m+i+1\right)e_{2}+e_{1}\right)\left(-\left(m+i+1\right)e_{2}-e_{1}\right)\text{,}$$

$$V_{4}=e_{2}\left(-e_{2}\right)\text{.}$$

We have $U,V_{1},\ldots ,V_{4}\in A\left(G\right),\left|U\right|=D\left(G\right),V_{1}\left(-V_{1}\right)V_{2}\left(-V_{2}\right)V_{3}V_{4}^{i}=\left(-U\right)U$, and thus 5+i∈L((−U)U) for i∈[0,mn−1], and therefore $\left[5,mn+4\right]\subset U_{M}\left(G\right)$.

*Case* 5. m≥6 even. We begin by showing that $4\in U_{M}\left(G\right)$. Set

$$U=e_{2}^{mn-1}{\left(ne_{2}+e_{1}\right)}^{\frac{m}{2}-2}{\left(-ne_{2}+e_{1}\right)}^{\frac{m}{2}}\left(\left(2n+1\right)e_{2}+e_{1}\right)e_{1}\text{,}$$

$$V_{1}={\left(ne_{2}+e_{1}\right)}^{\frac{m}{2}-2}{\left(ne_{2}-e_{1}\right)}^{\frac{m}{2}-1}\left(\left(2n+1\right)e_{2}+e_{1}\right)e_{2}^{n-1}\text{,}$$

$$V_{2}=\left(-ne_{2}+e_{1}\right)\left(-e_{1}\right){\left(-e_{2}\right)}^{mn-n}\text{.}$$

We have $U,\;V_{1},\;V_{2}\in A\left(G\right),\left|U\right|=D\left(G\right)$, $V_{1}\left(-V_{1}\right)V_{2}\left(-V_{2}\right)=\left(-U\right)U$, and thus $4\in L\left(\left(-U\right)U\right)\subset U_{M}\left(G\right)$.

Next we show that $5\in U_{M}\left(G\right)$. Set

$$U=e_{2}^{mn-1}{\left(ne_{2}+e_{1}\right)}^{\frac{m-2}{2}-1}{\left(-ne_{2}+e_{1}\right)}^{\frac{m-2}{2}}\left(-2ne_{2}+e_{1}\right)\left(\left(3n+1\right)e_{2}+e_{1}\right)e_{1}\text{,}$$

$$V_{1}=e_{2}^{mn-n}\left(ne_{2}+e_{1}\right)\left(-e_{1}\right)\text{,}$$

$$V_{2}={\left(-e_{2}\right)}^{mn-n}\left(-2ne_{2}+e_{1}\right)\left(ne_{2}-e_{1}\right)\text{,}$$

$$V_{3}={\left(ne_{2}+e_{1}\right)}^{\frac{m-2}{2}-2}{\left(ne_{2}-e_{1}\right)}^{\frac{m-2}{2}-2}\left(\left(3n+1\right)e_{2}+e_{1}\right)\left(2ne_{2}-e_{1}\right)e_{2}^{n-1}\text{,}$$

$$V_{4}={\left(-ne_{2}+e_{1}\right)}^{\frac{m-2}{2}-1}{\left(-ne_{2}-e_{1}\right)}^{\frac{m-2}{2}-1}\left(-\left(3n+1\right)e_{2}-e_{1}\right)e_{1}{\left(-e_{2}\right)}^{n-1}\text{,}$$

$$V_{5}=\left(-ne_{2}+e_{1}\right)\left(ne_{2}-e_{1}\right)\text{.}$$

We have $U,\;V_{1},\ldots ,V_{5}\in A\left(G\right),\left|U\right|=D\left(G\right),V_{1}V_{2}V_{3}V_{4}V_{5}=\left(-U\right)U$, and thus $5\in L\left(\left(-U\right)U\right)\subset U_{M}\left(G\right)$. It remains to show $\left[6,mn-1\right]\subset U_{M}\left(G\right)$.

For i∈[0,mn−1], we set

$$U=e_{2}^{mn-1}{\left(e_{2}+e_{1}\right)}^{\frac{m-6}{2}}\left(\left(m+i+2\right)e_{2}+e_{1}\right)\left(\left(3-m\right)e_{2}+e_{1}\right){\left(-e_{2}+e_{1}\right)}^{\frac{m-6}{2}}\left(\left(-1-i\right)e_{2}+e_{1}\right)\left(-3e_{2}+e_{1}\right)e_{1}^{2}\text{,}$$

$$V_{1}={\left(e_{2}+e_{1}\right)}^{\frac{m-6}{2}}\left(\left(3-m\right)e_{2}+e_{1}\right){\left(e_{2}-e_{1}\right)}^{\frac{m-6}{2}}\left(3e_{2}-e_{1}\right)\text{,}$$

$$V_{2}=e_{2}^{mn-1-i}e_{1}\left(\left(1+i\right)e_{2}-e_{1}\right)\text{,}$$

$$V_{3}=\left(\left(m+i+2\right)e_{2}+e_{1}\right)\left(-\left(m+i+2\right)e_{2}-e_{1}\right)\text{,}$$

$$V_{4}=e_{2}\left(-e_{2}\right)\text{,}$$

$$V_{5}=e_{1}\left(-e_{1}\right)\text{.}$$

We have $U,\;V_{1},\ldots ,V_{5}\in A\left(G\right),\left|U\right|=D\left(G\right)$, $V_{1}\left(-V_{1}\right)V_{2}\left(-V_{2}\right)V_{3}V_{4}^{i}V_{5}=\left(-U\right)U$, and thus 6+i∈L((−U)U) for i∈[0,mn−1], and therefore $\left[6,mn+5\right]\subset U_{M}\left(G\right)$. □

## 4 Products of two atoms in Krull monoids with class group of rank greater than two

We start with a simple technical lemma.

Lemma 4.1 *Let* $G=G_{1}⊕G_{2}$ *with* $G_{1},G_{2}\subset G$ *non-trivial subgroups satisfying* $d^{*}\left(G_{1}\right)+d^{*}\left(G_{2}\right)=d^{*}\left(G\right)$ *and suppose that* $U_{1}\in A\left(G_{1}\right)$ *with* $\left|U_{1}\right|=D^{*}\left(G_{1}\right)$ *and* $l\in L\left(\left(-U_{1}\right)U_{1}\right)\cap \left[2,d^{*}\left(G_{1}\right)\right]$ *. Then there exists a* U∈A(G) *with* $\left|U\right|=D^{*}\left(G\right)$ *such that* l∈L((−U)U) *. In particular,*

$$U_{\left\{2,D^{*}\left(G_{1}\right)\right\}}\left(G_{1}\right)\setminus \left\{D^{*}\left(G_{1}\right)\right\}\subset U_{\left\{2,D^{*}\left(G\right)\right\}}\left(G\right)\text{.}$$

Proof By hypothesis, there are $V_{1},\ldots ,V_{l}\in A\left(G_{1}\right)$ such that

$$\left(-U_{1}\right)U_{1}=V_{1}\cdot \ldots \cdot V_{l}\text{.}$$

Moreover, by re-indexing as need be, we can assume $\left|V_{1}\right|=\cdots =\left|V_{k}\right|=2$ and $\left|V_{i}\right|\geq 3$ for all i∈[k+1,l], where k∈[0,l]. Since $l\leq d^{*}\left(G_{1}\right)<D^{*}\left(G_{1}\right)=\left|U_{1}\right|$, it follows that k<l. Clearly, there is an S∈F(G) such that $V_{k+1}\cdot \ldots \cdot V_{l}=\left(-S\right)S$. Furthermore, there are g∈G and $S_{l-1},S_{l}\in F\left(G\right)$ such that w.l.o.g. $V_{l}=gS_{l}$ and $V_{l-1}=\left(-g\right)S_{l-1}$.

We choose a basis $\left(e_{1},\ldots ,e_{r}\right)$ of $G_{2}$ such that $d^{*}\left(G_{2}\right)=\sum_{i=1}^{r}\left(n_{i}-1\right)$, where $n_{i}=\mathrm{ord}\left(e_{i}\right)$ for i∈[1,r], and set $e_{0}=e_{1}+\cdots +e_{r}$. We define

$$T=e_{1}^{n_{1}-1}\cdot \ldots \cdot e_{r}^{n_{r}-1}\text{,}\;V_{l}^{\prime }=T\left(e_{0}+g\right)S_{l}\text{,}\;V_{l-1}^{\prime }=\left(-T\right)\left(-e_{0}-g\right)S_{l-1}\text{,}\;\text{and}\;U=T\left(e_{0}+g\right)g^{-1}U_{1}\text{.}$$

Then $V_{l-1}^{\prime },V_{l}^{\prime },U\in A\left(G\right)$, and it follows that

$$\left(-U\right)U=V_{1}\cdot \ldots \cdot V_{l-2}V_{l-1}^{\prime }V_{l}^{\prime }$$

and

$$\left|U\right|=\left(\left|U_{1}\right|-1\right)+\left|T\right|+1=d^{*}\left(G_{1}\right)+d^{*}\left(G_{2}\right)+1=d^{*}\left(G\right)+1=D^{*}\left(G\right)\text{.}$$

The desired inclusion is an immediate consequence. □

Theorem 4.2 *Let* H *be a Krull monoid with class group* $G≅C_{n_{1}}⊕\cdots ⊕C_{n_{r}}$ *, where* $r\geq 3,n_{r-1}\geq 3$ *and* $1<n_{1}|\ldots |n_{r}$ *, and suppose that every class contains a prime divisor. Then* $U_{\left\{2,D^{*}\left(G\right)\right\}}\left(H\right)⊃\left[2,D^{*}\left(G\right)\right]$ *.*

Proof By Lemma 2.1, it suffices to prove the assertion for the monoid B(G) where $G=C_{n_{1}}⊕\cdots ⊕C_{n_{r}}$. We start with the following assertion.

**A1.** Let r=3. Then there exists a U∈A(G) such that $\left\{2,3,D^{*}\left(G\right)\right\}\subset L\left(\left(-U\right)U\right)$.

Suppose that **A1** holds. We set $M=\left\{2,D^{*}\left(G\right)\right\}$ and proceed by induction on r. If r=3, then Theorem 3.5, **A1**, and Lemma 4.1 show that $\left[2,d^{*}\left(C_{n_{2}}⊕C_{n_{3}}\right)\right]\subset U_{M}\left(G\right)$, and Lemma 3.3 implies that $\left[d^{*}\left(C_{n_{1}}\right)+n_{3},D^{*}\left(G\right)\right]\subset U_{M}\left(G\right)$. Suppose that r≥4 and observe that, for $G^{\prime }=C_{n_{2}}⊕\cdots ⊕C_{n_{r}}$, the induction hypothesis implies that $\left[2,D^{*}\left(G^{\prime }\right)\right]\subset U_{\left\{2,D^{*}\left(G^{\prime }\right)\right\}}\left(G^{\prime }\right)$. Then by Lemma 4.1 we have $\left[2,d^{*}\left(G^{\prime }\right)\right]\subset U_{M}\left(G\right)$, and Lemma 3.3 implies that $\left[d^{*}\left(C_{n_{1}}⊕\cdots ⊕C_{n_{r-2}}\right)+n_{r},D^{*}\left(G\right)\right]\subset U_{M}\left(G\right)$.

Thus it remains to prove **A1**. To do so, we need two auxiliary assertions.

**A2.** Let m,n∈N with m≥5 odd and m∣n. Then there is a sequence $S\in F\left(C_{n}\right)$ of length |S|=m−1 with a decomposition $S=S_{1}S_{2}s$ with $S_{1},\;S_{2}\in F\left(C_{n}\right),\left|S_{1}\right|=\frac{m-1}{2},\left|S_{2}\right|=\frac{m-3}{2}$, and $s\in C_{n}$ such that the following conditions are fulfilled:
  • $2\sigma \left(S_{1}\right)=s$ and σ(S)=0;
  • Any zero-sum subsequence of S does not have the same number of terms from $S_{1}$ as from $S_{2}s$ unless it is the entire sequence or trivial.
**A3.** Let m,n∈N with m≥8 even, m∣n, and let $e\in C_{n}$ have order n. Then there is a sequence $S\in F\left(C_{n}\right)$ of length |S|=m−2 with a decomposition $S=s_{1}s_{2}S_{1}S_{2}$ with $S_{1},\;S_{2}\in F\left(C_{n}\right),\left|S_{1}\right|=\frac{m}{2}-1,\left|S_{2}\right|=\frac{m}{2}-3$, and $s_{1},\;s_{2}\in C_{n}$ such that the following conditions are fulfilled:
  • $\sigma \left(s_{1}s_{2}S_{1}S_{2}\right)=-e$;
  • $2\left(s_{1}+s_{2}\right)-\sigma \left(S_{1}\right)+\sigma \left(S_{2}\right)=-e$;
  • Any subsequence from $s_{1}s_{2}S_{1}S_{2}$ with sum 0 or −e does not have the same number of terms from $s_{1}s_{2}S_{2}$ as from $S_{1}$ unless it is the entire sequence or trivial.

Proof of A2 We choose an element $e\in C_{n}$ with ord(e)=n and distinguish two cases.

*Case* 1. n is odd.

We set

$$S_{1}=\left(\frac{n+1}{2}e\right)0^{\frac{m-3}{2}}\text{,}\;S_{2}=e^{\frac{m-5}{2}}\left(\frac{n-m+2}{2}e\right)\text{,}\;\text{and}\;s=e\text{.}$$

Now we find $\left|S_{1}\right|=\frac{m-1}{2},\left|S_{2}\right|=\frac{m-3}{2}$, and |S|=m−1. Next we show the two additional conditions.

We find

$$2\sigma \left(S_{1}\right)=2\cdot \frac{n+1}{2}e=\left(n+1\right)e=e=s\;\text{and}\;\sigma \left(S\right)=\left(\frac{n+1}{2}+\frac{n-3}{2}+1\right)e=0\text{,}$$

and thus the first condition is satisfied. Next we calculate the sumsets of $S_{2}s$ and $S_{1}$. We have

$$\Sigma \left(S_{2}s\right)=\left\{e,\ldots ,\frac{m-3}{2}e\right\}\cup \left\{\frac{n-m+2}{2}e,\ldots ,\frac{n-1}{2}e\right\}\;\text{and}\;\Sigma \left(S_{1}\right)=\left\{0,\frac{n+1}{2}e\right\}\text{.}$$

If T∣S is a non-trivial zero-sum subsequence with a decomposition $T=T_{1}T_{2}$, where $1\neq T_{1}|S_{1},1\neq T_{2}|S_{2}s$, and $\left|T_{1}\right|=\left|T_{2}\right|$, then we find $S_{2}s\left(\frac{n+1}{2}e\right)|T$, and thus $T_{2}=S_{2}s,T_{1}=S_{1}$, and T=S.

*Case* 2. n is even.

Note that m odd and n even implies that 2m≤n. We set

$$S_{1}=\left(\frac{n+2}{2}e\right)0^{\frac{m-3}{2}}\text{,}\;S_{2}={\left(2e\right)}^{\frac{m-5}{2}}\left(\left(\frac{n}{2}-m+2\right)e\right)\text{,}\;\text{and}\;s=2e\text{.}$$

Now we again find $\left|S_{1}\right|=\frac{m-1}{2},\left|S_{2}\right|=\frac{m-3}{2}$, and |S|=m−1. Next we show the two additional conditions.

We find

$$2\sigma \left(S_{1}\right)=2\cdot \frac{n+2}{2}e=2e=s\;\text{and}\;\sigma \left(S\right)=\left(\frac{n}{2}+1+\frac{n}{2}-3+2\right)e=0\text{,}$$

and thus the first condition is satisfied. Next we calculate the sumsets of $S_{2}s$ and $S_{1}$. We have

$$\Sigma \left(S_{1}\right)=\left\{0,\frac{n+2}{2}e\right\}\;\text{and}$$

$$\Sigma \left(S_{2}s\right)=\left\{2e,4e,\ldots ,\left(m-3\right)e\right\}\cup \left\{\left(\frac{n}{2}-m+2\right)e,\left(\frac{n}{2}-m+4\right)e,\ldots ,\left(\frac{n}{2}-1\right)e\right\}\text{.}$$

If T∣S is a non-trivial zero-sum subsequence with a decomposition $T=T_{1}T_{2}$, where $1\neq T_{1}|S_{1},1\neq T_{2}|S_{2}s$, and $\left|T_{1}\right|=\left|T_{2}\right|$, then we find $S_{2}s\left(\frac{n+1}{2}e\right)|T$, and thus $T_{2}=S_{2}s,T_{1}=S_{1}$, and T=S. □

Proof of A3 Let $e\in C_{n}$ with ord(e)=n. We set

$$S_{1}={\left(2e\right)}^{\frac{m}{2}-2}\left(\left(4-m\right)e\right)\text{,}\;S_{2}=0^{\frac{m}{2}-4}\left(-e\right)\text{,}\;\text{and}\;s_{1}=s_{2}=0\text{.}$$

Now we find $\left|S_{1}\right|=\frac{m}{2}-1,\left|S_{2}\right|=\frac{m}{2}-3$, and |S|=m−2. Furthermore, we have

$$\sigma \left(s_{1}s_{2}S_{1}S_{2}\right)=-e\;\text{and}\;2\left(s_{1}+s_{2}\right)-\sigma \left(S_{1}\right)+\sigma \left(S_{2}\right)=-e\text{,}$$

and thus the first two conditions are fulfilled. Next we calculate the sumsets of $s_{1}s_{2}S_{2}$ and $S_{1}$. We have

$$\Sigma \left(S_{1}\right)=\left\{2e,4e,\ldots ,\left(m-4\right)e\right\}\cup \left\{-\left(m-4\right)e,-\left(m-6\right)e,\ldots ,-2e,0\right\}\;\text{and}\;\Sigma \left(s_{1}s_{2}S_{2}\right)=\left\{0,-e\right\}\text{.}$$

Since $S_{1}$ has no proper non-trivial zero-sum subsequence, we find that the third condition is satisfied. □

Proof of A1 If $n_{1}\in \left[3,4\right]$ and $G_{1}=C_{n_{1}}⊕C_{n_{2}}$, then $3\in U_{\left\{2,D^{*}\left(G_{1}\right)\right\}}\left(G_{1}\right)$ by Theorem 3.5. Since $3\leq d^{*}\left(G_{1}\right)$, Lemma 4.1 implies the assertion. Thus we may assume that $n_{1}\geq 5$.

Let $\left(e_{1},e_{2},e_{3}\right)$ be a basis of G with $\mathrm{ord}\left(e_{i}\right)=n_{i}$, and let $p_{i}:G\to \langle e_{i}\rangle$ denote the canonical projection for every i∈[1,3]. For an element $g=a_{1}e_{1}+a_{2}e_{2}+a_{3}e_{3}\in G$, with $a_{i}\in \left[0,n_{i}-1\right]$ for all i∈[1,3], we set

$$\left(\begin{array}{c} a_{1}\\ a_{2}\\ a_{3} \end{array}\right)=a_{1}e_{1}+a_{2}e_{2}+a_{3}e_{3}\text{.}$$

Moreover, for an element $a_{1}e_{1}\in \langle e_{1}\rangle$ with $a_{1}\in \left[0,n_{1}-1\right]$, a sequence $S_{2}\in F\left(\langle e_{2}\rangle \right)$ and a sequence $S_{3}\in F\left(\langle e_{3}\rangle \right)$ with $\left|S_{2}\right|=\left|S_{3}\right|$, we denote by

$$S=\left(\begin{array}{c} a_{1}\\ S_{2}\\ S_{3} \end{array}\right)\in F\left(G\right)\text{ a sequence satisfying }\left\{ \begin{array}{c} p_{1}\left(S\right)={\left(a_{1}e_{1}\right)}^{\left|S_{2}\right|}\\ p_{2}\left(S\right)=S_{2}\\ p_{3}\left(S\right)=S_{3}\text{.} \end{array}\right.$$

Now we distinguish three cases based on $n_{1}$.

*Case* 1. $n_{1}=6$.

In this particular case, we set

$$U=e_{2}^{n_{2}-1}e_{3}^{n_{3}-1}\left(e_{1}-e_{3}\right)\left(e_{1}+2e_{2}-e_{3}\right)\left(e_{1}+3e_{2}+3e_{3}\right)\left(e_{1}-2e_{2}-2e_{3}\right)\left(e_{1}-e_{2}\right)\left(e_{1}-e_{2}+2e_{2}\right)\text{,}$$

$$V_{1}={\left(-e_{2}\right)}^{n_{2}-4}e_{3}^{n_{3}-4}\left(e_{1}-e_{2}\right)\left(e_{1}-e_{2}+2e_{3}\right)\left(-e_{1}+e_{3}\right)\left(-e_{1}-2e_{2}+e_{3}\right)\text{,}$$

$$V_{2}={\left(-e_{2}\right)}^{3}{\left(-e_{3}\right)}^{n_{3}-1}\left(e_{1}+3e_{2}+3e_{3}\right)\left(e_{1}-2e_{2}-2e_{3}\right)\left(-e_{1}+e_{2}\right)\left(-e_{1}+e_{2}-2e_{3}\right)\text{,}$$

$$V_{3}=e_{3}^{3}e_{2}^{n_{2}-1}\left(e_{1}-e_{3}\right)\left(e_{1}+2e_{2}-e_{3}\right)\left(-e_{1}-3e_{2}-3e_{3}\right)\left(-e_{1}+2e_{2}+2e_{3}\right)\text{,}$$

and we find $U,\;V_{1},\;V_{2},\;V_{3}\in A\left(G\right),\left|U\right|=D^{*}\left(G\right)$, and $V_{1}V_{2}V_{3}=\left(-U\right)U$, and thus $\left\{2,3,D^{*}\left(G\right)\right\}\subset L\left(\left(-U\right)U\right)$.

*Case* 2. $n_{1}\geq 8$ even.

Let $S=\left(s_{1}e_{2}\right)\left(s_{2}e_{2}\right)S_{1}S_{2}\in F\left(\langle e_{2}\rangle \right)$ with $s_{1},s_{2}\in \left[0,n_{2}-1\right]$ and $T=\left(t_{1}e_{3}\right)\left(t_{2}e_{3}\right)T_{1}T_{2}\in F\left(\langle e_{3}\rangle \right)$ with $t_{1},t_{2}\in \left[0,n_{3}-1\right]$ be two sequences of length $\left|S\right|=\left|T\right|=n_{1}-2$ fulfilling the conditions from A3. Now we set

$$U={\left(\begin{array}{c} 0\\ 1\\ 0 \end{array}\right)}^{n_{2}-1}{\left(\begin{array}{c} 0\\ 0\\ 1 \end{array}\right)}^{n_{3}-1}\left(\begin{array}{c} 1\\ s_{1}\\ 1+t_{2} \end{array}\right)\left(\begin{array}{c} 1\\ s_{2}\\ 1+t_{1} \end{array}\right)\left(\begin{array}{c} 1\\ s_{1}+1\\ t_{1} \end{array}\right)\left(\begin{array}{c} 1\\ s_{2}+1\\ t_{2} \end{array}\right)\left(\begin{array}{c} 1\\ -S_{1}\\ -T_{1} \end{array}\right)\left(\begin{array}{c} 1\\ S_{2}\\ T_{2} \end{array}\right)\text{,}$$

$$V_{1}={\left(\begin{array}{c} 0\\ -1\\ 0 \end{array}\right)}^{n_{2}-2}{\left(\begin{array}{c} 0\\ 0\\ 1 \end{array}\right)}^{n_{3}-2}\left(\begin{array}{c} 1\\ s_{1}\\ 1+t_{2} \end{array}\right)\left(\begin{array}{c} 1\\ s_{2}\\ 1+t_{1} \end{array}\right)\left(\begin{array}{c} -1\\ -s_{1}-1\\ -t_{1} \end{array}\right)\left(\begin{array}{c} -1\\ -s_{2}-1\\ -t_{2} \end{array}\right)\text{,}$$

$$V_{2}=\left(\begin{array}{c} 0\\ -1\\ 0 \end{array}\right){\left(\begin{array}{c} 0\\ 0\\ -1 \end{array}\right)}^{n_{3}-1}\left(\begin{array}{c} -1\\ -s_{1}\\ -1-t_{2} \end{array}\right)\left(\begin{array}{c} -1\\ -s_{2}\\ -1-t_{1} \end{array}\right)\left(\begin{array}{c} 1\\ -S_{1}\\ -T_{1} \end{array}\right)\left(\begin{array}{c} -1\\ -S_{2}\\ -T_{2} \end{array}\right)\text{,}$$

$$V_{3}={\left(\begin{array}{c} 0\\ 1\\ 0 \end{array}\right)}^{n_{2}-1}\left(\begin{array}{c} 0\\ 0\\ 1 \end{array}\right)\left(\begin{array}{c} 1\\ s_{1}+1\\ t_{1} \end{array}\right)\left(\begin{array}{c} 1\\ s_{2}+1\\ t_{2} \end{array}\right)\left(\begin{array}{c} 1\\ S_{2}\\ T_{2} \end{array}\right)\left(\begin{array}{c} -1\\ S_{1}\\ T_{1} \end{array}\right)\text{,}$$

and we find $\left|U\right|=D^{*}\left(G\right)$ and $V_{1}V_{2}V_{3}=\left(-U\right)U$. Since S and T have the special properties from A3, we have $U,\;V_{1},\;V_{2},\;V_{3}\in A\left(G\right)$, and thus $\left\{2,3,D^{*}\left(G\right)\right\}\subset L\left(\left(-U\right)U\right)$.

*Case* 3. $n_{1}\geq 5$ odd.

Let $S=S_{1}S_{2}\left(se_{2}\right)\in F\left(\langle e_{2}\rangle \right)$ with $s\in \left[0,n_{2}-1\right]$ and $T=T_{1}T_{2}\left(te_{3}\right)\in F\left(\langle e_{3}\rangle \right)$ with $t\in \left[0,n_{3}-1\right]$ be two sequences of length $\left|S\right|=\left|T\right|=n_{1}-1$ fulfilling the conditions from A2. Now we set

$$U={\left(\begin{array}{c} 0\\ 1\\ 0 \end{array}\right)}^{n_{2}-1}{\left(\begin{array}{c} 0\\ 0\\ 1 \end{array}\right)}^{n_{3}-1}\left(\begin{array}{c} 1\\ S_{1}\\ T_{1} \end{array}\right)\left(\begin{array}{c} 1\\ -S_{2}\\ -T_{2} \end{array}\right)\left(\begin{array}{c} 1\\ 1-s\\ -t \end{array}\right)\left(\begin{array}{c} 1\\ -s\\ 1-t \end{array}\right)\text{,}$$

$$V_{1}={\left(\begin{array}{c} 0\\ 1\\ 0 \end{array}\right)}^{n_{2}-1}{\left(\begin{array}{c} 0\\ 0\\ -1 \end{array}\right)}^{n_{3}-1}\left(\begin{array}{c} 1\\ 1-s\\ -t \end{array}\right)\left(\begin{array}{c} -1\\ s\\ t-1 \end{array}\right)\text{,}$$

$$V_{2}={\left(\begin{array}{c} 0\\ -1\\ 0 \end{array}\right)}^{n_{2}-1}\left(\begin{array}{c} 1\\ S_{1}\\ T_{1} \end{array}\right)\left(\begin{array}{c} -1\\ S_{2}\\ T_{2} \end{array}\right)\left(\begin{array}{c} -1\\ s-1\\ t \end{array}\right)\text{,}$$

$$V_{3}={\left(\begin{array}{c} 0\\ 0\\ 1 \end{array}\right)}^{n_{3}-1}\left(\begin{array}{c} 1\\ -S_{2}\\ -T_{2} \end{array}\right)\left(\begin{array}{c} 1\\ -s\\ 1-t \end{array}\right)\left(\begin{array}{c} -1\\ -S_{1}\\ -T_{1} \end{array}\right)\text{,}$$

and we find $\left|U\right|=D^{*}\left(G\right)$ and $V_{1}V_{2}V_{3}=\left(-U\right)U$. Since S and T have the special properties from A2, we have $U,\;V_{1},\;V_{2},\;V_{3}\in A\left(G\right)$, and thus $\left\{2,3,D^{*}\left(G\right)\right\}\subset L\left(\left(-U\right)U\right)$. □

## 5 Arithmetical characterizations of class groups

Two reduced Krull monoids H and $H^{\prime }$ are isomorphic if and only if there is a group isomorphism $\Phi :C\left(H\right)\to C\left(H^{\prime }\right)$ such that, for every class g∈C(H), the number of primes in g equals the number of primes in the class $\Phi \left(g\right)\in C\left(H^{\prime }\right)$[17, Theorem 2.5.4]. This justifies the classical philosophy in algebraic number theory that the class group of a ring of integers completely determines its arithmetic. Initiated by Narkiewicz in the 1970s, the reverse question—to what extent do arithmetical phenomena characterize the class group—has been tackled and has received a wide variety of different arithmetical characterizations (for an overview, see [17, Sections 7.1 and 7.2]). Sets of lengths are the most investigated invariant in factorization theory, and the problem of whether the system of all sets of lengths L(H)=L(G) is characteristic for the class group G has received special attention. An affirmative answer—that is, if $L\left(G\right)=L\left(G^{\prime }\right)$, then G and $G^{\prime }$ are isomorphic—was given so far for cyclic groups, groups of the form $C_{n}⊕C_{n}$ and others (see  [28], [14], [30], [29], [33]). In this section, we use our results on $U_{\left\{2,D^{*}\left(G\right)\right\}}\left(G\right)$ to obtain some characterization results for groups of rank two (see Theorem 5.6).

To introduce the necessary concepts, let G be a finite abelian group and $S=g_{1}\cdot \ldots \cdot g_{l}$ a sequence over G. Then

$$k\left(S\right)=\overset{l}{\underset{i=1}{\sum }}\frac{1}{\mathrm{ord}\left(g_{i}\right)}\;\in Q\;\text{resp. }K\left(G\right)=\max\left\{k\left(S\right)|S\in A\left(G\right)\right\}$$

denote the *cross number* of S (resp. the *cross number* of G; for recent progress on K(G), see  [15], [20], [21]).

Let  d,l∈N  and   $M\in N_{0}$. A subset   L⊂Z  is called an *almost arithmetical progression* (AAP) with *difference* d, *length* l, and *bound* M if

$$L=y+\left(L^{\prime }\cup L^{*}\cup L^{\prime \prime }\right)\subset y+dZ\text{,}$$

where $y\in Z,L^{*}=\left\{\nu d|\nu \in \left[0,l\right]\right\}$ is an arithmetical progression with difference d and length $l,L^{\prime }\subset \left[-M,-1\right]$, and $L^{\prime \prime }\subset \max L^{*}+\left[1,M\right]$.

We set

$$\Delta^{*}\left(G\right)=\left\{\min\Delta \left(G_{0}\right)|G_{0}\subset G\;\text{with}\;\Delta \left(G_{0}\right)\neq 0̸\right\}$$

and let $\Delta_{1}\left(G\right)\subset \Delta \left(G\right)$ denote the set of all d∈N with the following property:

For every k∈N, there exists some L∈L(G) which is an AAP with difference d and length l≥k.

The sets $\Delta^{*}\left(G\right)$ and $\Delta_{1}\left(G\right)$ have been studied by Chapman, Geroldinger, Hamidoune, Plagne, Schmid, Smith and others (see, for example,  [18], [4], [26] and  [17, Section 6.8] for some basic information).

A subset $G_{0}\subset G$ is called an LCN-set if k(A)≥1 for all $A\in A\left(G_{0}\right)$. Moreover, we define

$$m\left(G\right)=\max\left\{\min\Delta \left(G_{0}\right)|G_{0}\subset G\;\text{is an LCN-set with}\;\Delta \left(G_{0}\right)\neq 0̸\right\}\text{,}$$

using the convention that max0̸=0.

Lemma 5.1 *Let* G *be a finite abelian group with* |G|≥3 *.*

1. $m\left(G\right)\leq \max\left\{r^{*}\left(G\right)-1,K\left(G\right)-1\right\}$ *.*
2. $\max\Delta^{*}\left(G\right)=\max\left\{\exp\left(G\right)-2,m\left(G\right)\right\}$ *.*

Proof For item 1, see  [29, Proposition 3.6], while item 2 is a consequence of  [17, Theorem 6.8.10]. □

Lemma 5.2 *Let* G *and* $G^{\prime }$ *be finite abelian groups with* $\left|G^{\prime }\right|\geq 3$ *such that* $L\left(G\right)=L\left(G^{\prime }\right)$ *. Then we have* $D\left(G\right)=D\left(G^{\prime }\right),\;\Delta_{1}\left(G\right)=\Delta_{1}\left(G^{\prime }\right)$ *and* $\max\Delta^{*}\left(G\right)=\max\Delta^{*}\left(G^{\prime }\right)$ *. Moreover, we have*

$$\left\{d\in \Delta^{*}\left(G\right)\;|\;d>\frac{\max\Delta^{*}\left(G\right)}{2}\right\}=\left\{d\in \Delta^{*}\left(G^{\prime }\right)\;|\;d>\frac{\max\Delta^{*}\left(G^{\prime }\right)}{2}\right\}\text{.}$$

Proof The first three statements are proved in  [17, Proposition 7.3.1]. Since

$$\Delta^{*}\left(G\right)\subset \Delta_{1}\left(G\right)\subset \left\{d_{1}\in \Delta \left(G\right)|d_{1}\;\text{divides some}\;d\in \Delta^{*}\left(G\right)\right\}$$

by  [17, Corollary 4.3.16], the moreover statement follows from the first assertions. □

Lemma 5.3 *Let*$G=C_{2}^{s}⊕\overset{˜}{G}$*, where*$s\in N_{0}$*and*$\overset{˜}{G}\subset G$*is a subgroup which has no direct summand isomorphic to*$C_{2}$*. Then*$d^{*}\left(G\right)\geq s+2r^{*}\left(\overset{˜}{G}\right)$*, and equality holds if and only if*G*is an elementary*  2*-group or an elementary*  3*-group.*

Proof By  [22, Lemma 4.1], we have

$$d^{*}\left(G\right)\geq d^{*}\left(C_{2}^{s}\right)+d^{*}\left(\overset{˜}{G}\right)=s+d^{*}\left(\overset{˜}{G}\right)\text{.}$$

We choose a basis $\left(e_{1},\ldots ,e_{t}\right)$ of $\overset{˜}{G}$ with $t=r^{*}\left(\overset{˜}{G}\right)$ and $\mathrm{ord}\left(e_{i}\right)=q_{i}$ prime powers for all i∈[1,t]. Moreover, we suppose that $q_{1}\leq \ldots \leq q_{t}$, and by assumption we get $2<q_{1}$. Thus  [17, Proposition 5.1.7] implies that

$$d^{*}\left(\overset{˜}{G}\right)\geq \mathrm{ord}\left(e_{1}\right)+\cdots +\mathrm{ord}\left(e_{t}\right)-r\left(\overset{˜}{G}\right)\geq \overset{t}{\underset{i=1}{\sum }}\left(q_{i}-1\right)\geq 2t=2r^{*}\left(\overset{˜}{G}\right)\text{.}$$

Putting this all together, we obtain

$$d^{*}\left(G\right)\geq s+2r^{*}\left(\overset{˜}{G}\right)\text{.}$$

If G is an elementary 2-group, then $\overset{˜}{G}=0$ and $d^{*}\left(G\right)=s$. If G is an elementary 3-group, then $s=0,G=\overset{˜}{G}$ and $d^{*}\left(G\right)=2r^{*}\left(G\right)$.

Now suppose that G is neither an elementary 2-group nor an elementary 3-group. Then t≥1. If $q_{t}\geq 4$, then the previous argument implies that

$$d^{*}\left(\overset{˜}{G}\right)\geq \overset{t-1}{\underset{i=1}{\sum }}\left(q_{i}-1\right)+\left(q_{t}-1\right)\geq 2\left(t-1\right)+3=1+2r^{*}\left(\overset{˜}{G}\right)\text{,}$$

and hence $d^{*}\left(G\right)\geq s+1+2r^{*}\left(\overset{˜}{G}\right)$.

Suppose that $q_{t}=3$. Since G is not an elementary 3-group, it follows that s≥1. Therefore r=min{s,t}≥1 and

$$G=C_{2}^{s}⊕C_{3}^{t}≅C_{6}^{r}⊕C_{2}^{s-r}⊕C_{3}^{t-r}\text{.}$$

We again use  [22, Lemma 4.1] and infer that

$$d^{*}\left(G\right)\geq d^{*}\left(C_{6}^{r}\right)+d^{*}\left(C_{2}^{s-r}\right)+d^{*}\left(C_{3}^{t-r}\right)=5r+\left(s-r\right)+2\left(t-r\right)=s+2t+2r\geq s+2r^{*}\left(\overset{˜}{G}\right)+2\text{.}$$

 □

It is easy to verify that $L\left(C_{1}\right)=L\left(C_{2}\right)$ and that $L\left(C_{3}\right)=L\left(C_{2}⊕C_{2}\right)$[17, Section 7.3].

Proposition 5.4 *Let* G *and* $G^{\prime }$ *be finite abelian groups such that* $L\left(G\right)=L\left(G^{\prime }\right)$ *and suppose that* $\left\{G,G^{\prime }\right\}\neq \left\{C_{1},C_{2}\right\}$ *and* $\left\{G,G^{\prime }\right\}\neq \left\{C_{3},C_{2}^{2}\right\}$ *. If* $\min\left\{r\left(G\right),r\left(G^{\prime }\right)\right\}\leq 2$ *, then* $\exp\left(G\right)=\exp\left(G^{\prime }\right)$ *.*

Proof If G or $G^{\prime }$ is either cyclic or an elementary 2-group, then $G≅G^{\prime }$ by  [17, Theorem 7.3.3], and hence $\exp\left(G\right)=\exp\left(G^{\prime }\right)$. Suppose that neither G nor $G^{\prime }$ is cyclic or an elementary 2-group. We w.l.o.g. set $G^{\prime }=C_{m}⊕C_{mn}$ with m≥2 and n∈N. If m=2, then the assertion follows from  [13, Satz 4]. If n=1, then the assertion follows from  [29, Theorem 4.1]. So we may suppose that n>1 and m>2.

Lemma 5.2 and  [17, Corollary 6.8.11] imply that

$$\max\Delta^{*}\left(G\right)=\max\Delta^{*}\left(C_{m}⊕C_{mn}\right)=mn-2\;\text{and}\;D\left(G\right)=D\left(C_{m}⊕C_{mn}\right)=m+mn-1\text{,}$$

and thus, by Lemma 5.1.2, we get

mn−2=max{exp(G)−2,m(G)}.

Assume to the contrary that exp(G)−2<m(G)=mn−2. Then it follows from Lemma 5.1.1 that

$$\exp\left(G\right)-1\leq mn-2=m\left(G\right)\leq \max\left\{r^{*}\left(G\right)-1,K\left(G\right)-1\right\}\text{,}$$

and we distinguish two cases.

First, suppose that the maximum on the right hand side equals K(G)−1. Since D(G)≥2K(G) (which follows trivially from the definitions involved), we get

m+mn−1=D(G)≥2K(G)≥2(mn−1),

a contradiction.

Second, suppose that the maximum on the right hand side equals $r^{*}\left(G\right)-1$. Then

(5.1) $$\exp\left(G\right)-1\leq m\left(G\right)=mn-2\leq r^{*}\left(G\right)-1\text{.}$$

We set $G=C_{2}^{s}⊕\overset{˜}{G}$, where $s\in N_{0}$ and $\overset{˜}{G}\subset G$ is a subgroup which has no direct summand isomorphic to $C_{2}$. Then $r^{*}\left(G\right)=s+r^{*}\left(\overset{˜}{G}\right)$, and Lemma 5.3 implies that

$$mn+m-1=D\left(G\right)\geq d^{*}\left(G\right)+1\geq s+1+2r^{*}\left(\overset{˜}{G}\right)\text{.}$$

Therefore, using (5.1), we get

$$mn+m-1\geq s+1+2r^{*}\left(G\right)-2s\geq -s+1+2\left(mn-1\right)\text{,}$$

and hence s≥mn−m>0. Thus G is not an elementary 3-group. Repeating the above calculation with the sharper statement of Lemma 5.3, we get

$$mn+m-1\geq d^{*}\left(G\right)+1\geq s+2+2r^{*}\left(\overset{˜}{G}\right)\geq s+2+2r^{*}\left(G\right)-2s\geq -s+2mn\text{,}\;\text{and thus}$$

s≥mn−m+1.

Hence, using  [17, Corollary 6.8.3], we obtain

(5.2) $$\Delta^{*}\left(G\right)⊃\Delta^{*}\left(C_{2}^{s}\right)=\left[1,s-1\right]⊃\left[1,mn-m\right]\text{.}$$

Clearly, we have $\Delta^{*}\left(C_{m}⊕C_{mn}\right)\subset \Delta^{*}\left(C_{mn}⊕C_{mn}\right)$. Corollaries 3.7 and 3.8 in  [29] imply that

(5.3) $$\max\left(\Delta^{*}\left(C_{mn}⊕C_{mn}\right)\setminus \left\{mn-2,mn-3\right\}\right)=\left\lfloor \frac{mn}{2}\right\rfloor -1$$

and that (note n>1 and $m\left(C_{m}⊕C_{mn}\right)\leq m\left(C_{mn}⊕C_{mn}\right)\leq \left\lfloor mn/2\right\rfloor -1$)

(5.4) $$mn-3\notin \Delta^{*}\left(C_{m}⊕C_{mn}\right)\text{.}$$

By Lemma 5.2, we have

$$D≔\left\{d\in \Delta^{*}\left(G\right)\;|\;d>\frac{\max\Delta^{*}\left(G\right)}{2}\right\}=\left\{d\in \Delta^{*}\left(C_{m}⊕C_{mn}\right)\;|\;d>\frac{\max\Delta^{*}\left(C_{m}⊕C_{mn}\right)}{2}\right\}\text{.}$$

Thus, in view of $mn-m>\frac{mn-2}{2}=\frac{\max\Delta^{*}\left(G\right)}{2}$ and (5.2), we see that mn−m∈D. However, in view of $\left\lfloor mn/2\right\rfloor -1\leq \frac{\max\Delta^{*}\left(C_{m}⊕C_{mn}\right)}{2}$, (5.3), (5.4), we see that the only possible element of $\Delta^{*}\left(C_{m}⊕C_{mn}\right)$ that is larger than ⌊mn/2⌋−1—and thus the only possible element of $\Delta^{*}\left(C_{m}⊕C_{mn}\right)$ larger than $\frac{\max\Delta^{*}\left(C_{m}⊕C_{mn}\right)}{2}$—is mn−2. As a result, D⊂{mn−2}, which combined with mn−m∈D shows that mn−m=mn−2, contradicting m>2. □

There is a recent result due to Schmid [33, Proposition 5.2] which derives the conclusion of Proposition 5.4, namely that $\exp\left(G\right)=\exp\left(G^{\prime }\right)$, under a much weaker assumption. We decided to provide the proof of the special situation, because this is precisely what we need, and because the proof is simpler than that of the more general case.

Lemma 5.5 *Let* $n\in N_{\geq 2}$ *.*

1. *If* $G=C_{2}^{3}⊕C_{4n}$ *, then* {2,4n,4n+3}∈L(G) *.*
2. *If* $G=C_{4}⊕C_{4n}$ *, then* {2,4n,4n+3}∉L(G) *.*

Proof 1. Let $G=C_{2}^{3}⊕C_{4n}$ and let $\left(e_{1},e_{2},e_{3},e_{4}\right)$ be a basis of G with $\mathrm{ord}\left(e_{1}\right)=\mathrm{ord}\left(e_{2}\right)=\mathrm{ord}\left(e_{3}\right)=2$ and $\mathrm{ord}\left(e_{4}\right)=4n$. We set $e_{0}=e_{1}+\cdots +e_{4}$ and $U=e_{1}e_{2}e_{3}e_{4}^{4n-1}e_{0}$. Then U∈A(G) with |U|=4n+3, and we assert that L((−U)U)={2,4n,4n+3}. Clearly, we have {2,4n+3}⊂L((−U)U). If V∈A(G) with $V|\left(-U\right)U,e_{0}\in \mathrm{supp}\left(V\right)$ and $V\notin \left\{\left(-e_{0}\right)e_{0},U\right\}$, then $V=e_{0}e_{1}e_{2}e_{3}\left(-e_{4}\right)$ and $\left(-U\right)U=\left(-V\right)V{\left(\left(-e_{4}\right)e_{4}\right)}^{4n-2}$, which implies that L((−U)U)={2,4n,4n+3}.

2. Let $G=C_{4}⊕C_{4n}$ and assume to the contrary that {2,4n,4n+3}∈L(G). Since 4n+3=D(G), there exists some U∈A(G) with |U|=D(G)=4n+3 and L((−U)U)={2,4n,4n+3}. We aim to construct a V∈A(G) of length |V|∉{2,5,4n+3} with V∣(−U)U. Then Lemma 2.2 will imply that 2+|U|−|V|∈L((−U)U), contradicting that L((−U)U)={2,4n,4n+3}.

We will use Theorem 3.1 to describe the structure of U. If there exists a g∈G with ord(g)∉{2,4n} and $g^{\mathrm{ord}\left(g\right)-1}|U$, then we are done by Lemma 2.2.

Therefore, if U has Type I in Theorem 3.1, then U must have the form

$$U=e_{1}^{4n-1}\overset{4}{\underset{i=1}{\prod }}\left(x_{i}e_{1}+e_{2}\right)\text{,}$$

where $\left(e_{1},e_{2}\right)$ is a basis of G with $\mathrm{ord}\left(e_{2}\right)=4$ and $\mathrm{ord}\left(e_{1}\right)=4n$, and where $x_{i}\in \left[0,4n-1\right]$ with $x_{1}+\cdots +x_{4}\equiv 1\mathrm{mod}4n$. If $x_{i}\equiv x_{j}$ for all i,j∈[1,4], then $x_{1}+\cdots +x_{4}\equiv 4x_{1}≢1\mathrm{mod}4n$, a contradiction. Therefore we can w.l.o.g. assume $x_{1}≢x_{2}\mathrm{mod}4n$. Thus $x_{1}-x_{2}\equiv l\mathrm{mod}4n$ with l∈[1,4n−1]. But then $V={\left(-e_{1}\right)}^{l}\left(x_{1}e_{1}+e_{2}\right)\left(-x_{2}e_{1}-e_{2}\right)\in A\left(G\right)$ and $V^{\prime }-e_{1}^{4n-l}\left(x_{1}e_{1}+e_{2}\right)\left(-x_{2}e_{1}-e_{2}\right)\in A\left(G\right)$ are both atoms dividing U(−U) with length at least 3 and at most 4n−1+2. Thus we have found the desired length atom unless $l+2=\left|V\right|=5=\left|V^{\prime }\right|=4n-l+2$, which implies l=2n and 2n+2=5, which is easily seen to be a contradiction by reducing modulo 2. So we conclude that U must instead have type II in Theorem 3.1

Thus Theorem 3.1 shows that U has the form

$$U={\left(e_{1}+ye_{2}\right)}^{4s-1}e_{2}^{4\left(n-s\right)+\epsilon }\overset{4-\epsilon }{\underset{i=1}{\prod }}\left(-x_{i}e_{1}+\left(-x_{i}y+1\right)e_{2}\right)\text{,}$$

where $\left(e_{1},e_{2}\right)$ is a basis of G with $\mathrm{ord}\left(e_{1}\right)=4$ and $\mathrm{ord}\left(e_{2}\right)=4n$, where y∈[0,4n−1],ϵ∈[1,3],s∈[1,n−1], and where $x_{i}\in \left[1,3\right]$ with $x_{1}+\cdots +x_{4-\epsilon }=3$. Moreover, either s=1 or $4ye_{2}=4e_{2}$.

If $x_{i}=1$ for some i∈[1,4−ϵ], then $V=\left(e_{1}+ye_{2}\right)\left(-e_{2}\right)\left(-e_{1}+\left(-y+1\right)e_{2}\right)\in A\left(G\right)$ has length |V|=3 and divides (−U)U, as desired. Thus we may assume $x_{i}\geq 2$ for all i∈[1,4−ϵ], which in view of $x_{1}+\cdots +x_{4-\epsilon }=3$ implies ϵ=3 and $x_{1}=3$. As result, we have

$$U={\left(e_{1}+ye_{2}\right)}^{4s-1}e_{2}^{4\left(n-s\right)+3}\left(-3e_{1}+\left(-3y+1\right)e_{2}\right)\text{,}$$

Let 4y−1≡lmod4n with l∈[0,4n−1]. Note, since 4y−1≡−1mod4, that we actually have l∈[1,4n−1]. Thus, if s=1, then

$$e_{2}^{4n-l}\left(e_{1}+ye_{2}\right)\left(3e_{1}+\left(3y-1\right)e_{2}\right)\in A\left(G\right)\;\text{and}\;{\left(-e_{2}\right)}^{l}\left(e_{1}+ye_{2}\right)\left(3e_{1}+\left(3y-1\right)e_{2}\right)\in A\left(G\right)$$

are both atoms dividing U(−U) with length at least 3 and at most 4n−1+2, and we obtain a contradiction as we did when U had type I unless one of them has the desired length. Therefore we can assume s∈[2,n−1], in which case we have

(5.5) $$4ye_{2}=4e_{2}$$

per part (e) in Theorem 3.1.

Suppose that 4(n−s)+3≥4s−3. Then (5.5) ensures that

$$V^{\prime }={\left(e_{1}+ye_{2}\right)}^{4s-1}{\left(-e_{2}\right)}^{4s-3}\left(-3e_{1}+\left(-3y+1\right)e_{2}\right)\in B\left(G\right)$$

is a subsequence of (−U)U of length $\left|V^{\prime }\right|=8s-3\notin \left\{2,3,4,5,4n+3\right\}$ (in view of s≥2). If $V^{\prime }$ is an atom, then we have found the desired length zero-sum subsequence. Otherwise, there must be an atom V dividing $V^{\prime }$ with support $\mathrm{supp}\left(V\right)=\left\{e_{1}+ye_{2},\;-e_{2}\right\}$. Let $k=v_{e_{1}+ye_{2}}\left(V\right)$ and let $l=v_{-e_{2}}\left(V\right)$. By considering the $e_{1}$-coordinate of σ(V), we see that k≡0mod4. By then, considering the $e_{2}$-coordinate of σ(V) modulo 4, we conclude that l≡0mod4. Hence, since k,l≠0, it follows that |V|=k+l≥8 with |V|≡0≢4n+3mod4, and we have found the desired length zero-sum subsequence. So we may instead assume that 4(n−s)+3≤4s−4.

But now (5.5) ensures that

$$V^{\prime }={\left(e_{1}+ye_{2}\right)}^{4n-\left(4s-1\right)}{\left(-e_{2}\right)}^{4n-\left(4s-3\right)}\left(3e_{1}+\left(3y-1\right)e_{2}\right)\in B\left(G\right)$$

is a subsequence of (−U)U of length $\left|V^{\prime }\right|=8\left(n-s\right)+5\notin \left\{2,5,4n+3\right\}$ (in view of s∈[2,n−1]). If $V^{\prime }$ is an atom, then we have found the desired length zero-sum subsequence. Otherwise, there must be an atom V dividing $V^{\prime }$ with support $\mathrm{supp}\left(V\right)=\left\{e_{1}+ye_{2},\;-e_{2}\right\}$, and arguing as in the case 4(n−s)+3≥4s−3 shows that V has the desired length, completing the proof. □

Theorem 5.6 *Let* G *be a finite abelian group with* |G|≥4 *,* m,n∈N *with* $m^{2}n\geq 4$ *, and suppose that* $L\left(G\right)=L\left(C_{m}⊕C_{mn}\right)$ *.*

1. *If* $d\left(G\right)=d^{*}\left(G\right)$ *, then* $G≅C_{m}⊕C_{mn}$ *.*
2. *If* mn *is a power of a prime, then* $G≅C_{m}⊕C_{mn}$ *.*

Proof Proposition 5.4 implies that exp(G)=mn. Thus, if mn is a power of a prime, then G is a p-group and hence $d\left(G\right)=d^{*}\left(G\right)$. Therefore it suffices to prove the first statement. Suppose that $d\left(G\right)=d^{*}\left(G\right)$.

If m=1, then there are several proofs for the assertion (see [17, Theorem 7.3.3] or  [14, Corollary 5.3.3]). If m=2, then the assertion follows from  [13, Satz 4]. So we may suppose that m≥3.

Since exp(G)=mn, we set $G=G^{\prime }⊕C_{mn}$ for a subgroup $G^{\prime }\subset G$ with $\exp\left(G^{\prime }\right)|mn$. We observe that $r\left(G\right)=r\left(G^{\prime }\right)+1$ and, using Lemma 5.2, that

$$m+mn-1=D\left(C_{m}⊕C_{mn}\right)=D\left(G\right)=d^{*}\left(G\right)+1=d^{*}\left(G^{\prime }\right)+mn\text{.}$$

If $G^{\prime }$ is cyclic, then $d^{*}\left(G^{\prime }\right)=m-1$ implies that $G^{\prime }≅C_{m}$, and thus $G≅C_{m}⊕C_{mn}$.

Now we suppose that $G^{\prime }$ is non-cyclic and note that $r\left(G\right)=r\left(G^{\prime }\right)+1\geq 3$. If m=3, then

$$2+3n=D\left(C_{3}⊕C_{3n}\right)=D\left(G\right)=d^{*}\left(G^{\prime }\right)+3n\text{,}$$

which implies that $G^{\prime }≅C_{2}⊕C_{2}$ and that n is even. However, Theorem 5.3 in  [33] implies that $L\left(C_{2}⊕C_{2}⊕C_{3n}\right)\neq L\left(C_{3}⊕C_{3n}\right)$, a contradiction. Suppose that m=4. Then

$$3+4n=D\left(C_{4}⊕C_{4n}\right)=D\left(G\right)=d^{*}\left(G^{\prime }\right)+4n\text{,}$$

which implies that $G^{\prime }$ is isomorphic to $C_{2}^{3}$. Now Lemma 5.5 yields a contradiction.

Suppose that m≥5. By Theorem 3.5, we have

$$3\notin U_{\left\{2,D^{*}\left(C_{m}⊕C_{mn}\right)\right\}}\left(C_{m}⊕C_{mn}\right)=U_{\left\{2,D\left(C_{m}⊕C_{mn}\right)\right\}}\left(C_{m}⊕C_{mn}\right)=U_{\left\{2,D\left(G\right)\right\}}\left(C_{m}⊕C_{mn}\right)\text{,}$$

and by Theorem 4.2 we have $3\in U_{\left\{2,D^{*}\left(G\right)\right\}}\left(G\right)=U_{\left\{2,D\left(G\right)\right\}}\left(G\right)$, a contradiction. □
